# Novel Genes Critical for Hypoxic Preconditioning in Zebrafish Are Regulators of Insulin and Glucose Metabolism

**DOI:** 10.1534/g3.115.018010

**Published:** 2015-04-03

**Authors:** Tania Manchenkov, Martina P. Pasillas, Gabriel G. Haddad, Farhad B. Imam

**Affiliations:** * Division of Neonatology, University of California San Diego School of Medicine, La Jolla, California 92093; † Division of Respiratory Medicine, University of California San Diego School of Medicine, La Jolla, California 92093; ‡ Department of Pediatrics, University of California San Diego School of Medicine, La Jolla, California 92093; § Rady Children’s Hospital-San Diego, San Diego, California 92123

**Keywords:** hormesis, hypoxia-ischemia, metabolic state, preconditioning, stress tolerance

## Abstract

Severe hypoxia is a common cause of major brain, heart, and kidney injury in adults, children, and newborns. However, mild hypoxia can be protective against later, more severe hypoxia exposure via “hypoxic preconditioning,” a phenomenon that is not yet fully understood. Accordingly, we have established and optimized an embryonic zebrafish model to study hypoxic preconditioning. Using a functional genomic approach, we used this zebrafish model to identify and validate five novel hypoxia-protective genes, including *irs2*, *crtc3*, and *camk2g2*, which have been previously implicated in metabolic regulation. These results extend our understanding of the mechanisms of hypoxic preconditioning and affirm the discovery potential of this novel vertebrate hypoxic stress model.

Hypoxia is a major cause of organ injury in children and adults alike (Giordano 2005; Moskowitz *et al.* 2010). In newborns, severe hypoxia can cause hypoxic-ischemic encephalopathy, neurocognitive impairment, seizures, and cerebral palsy (du Plessis and Volpe 2002). In adults, hypoxic-ischemic injury from stroke or heart attack is a major cause of death and disability. Intriguingly, there exists a protective phenomenon, termed hypoxic preconditioning (hPC), that occurs when brief or mild exposure to hypoxia protects against a subsequent more severe hypoxia exposure. The protective effect of hPC has been demonstrated in multiple animal species and in organs such as the brain, heart, kidneys, and skeletal muscle (Azad *et al.* 2012; Chang and Bargmann 2008; Gidday 2006; Murry *et al.* 1986; Zhou *et al.* 2008). Despite multiple previous studies of hypoxia signaling in model organisms and in mammals, the molecular pathways responsible for the important protective effect of hPC remain incompletely understood (Azad and Haddad 2013; Chang and Bargmann 2008; Wacker *et al.* 2012). In this study, we introduce a novel *in vivo* embryonic model of hypoxic preconditioning in zebrafish and use it to discover novel genes contributing to the hypoxic stress response and to establishment of hPC.

We selected the embryonic zebrafish to study hypoxic stress for at least four reasons: the power of zebrafish genetics combined with ease of embryonic manipulation make zebrafish useful to study the effects of hypoxia on development (Mendelsohn *et al.* 2008; Padilla and Roth 2001); pathways important for hypoxia, such as hypoxia-inducible factor (HIF) and non-HIF networks, are conserved in the zebrafish (Mendelsohn and Gitlin 2008; Rytkonen *et al.* 2008; van Rooijen *et al.* 2009); zebrafish are particularly resistant to hypoxic stress (Rytkonen *et al.* 2007); and hypoxic preconditioning has been found to occur in adult zebrafish and, hence, possibly lends itself to systemic study during embryogenesis and larval life as well (Rees *et al.* 2001). In this study, we used genome-wide differential expression and a novel developmental hypoxia assay to identify and validate multiple uncharacterized hypoxia-regulated genes required for acute hypoxic stress protection and/or hPC. This functional genomic approach in zebrafish for hypoxia-protective genes identified novel genes, such as *btr01*, in addition to established genes not previously implicated in hypoxic stress buffering, such as *irs2* and *crtc3*, whose known functions are in glucose and lipid metabolism (Long *et al.* 2011; Song *et al.* 2010). Morpholino (MO) knockdown of these zebrafish genes resulted in increased developmental susceptibility to acute hypoxia and/or hPC and was rescued by mRNA coinjection. These studies in zebrafish provide new insight into the molecular and cellular mechanisms of acute hypoxic stress tolerance and hPC, including a molecular connection between hypoxic preconditioning and insulin/glucose signaling.

## Materials and Methods

### Zebrafish husbandry and strains

Zebrafish from the TL/AB strain were maintained using standard procedures and developmental stages were determined as previously described (Kimmel *et al.* 1995). Embryos were raised at 28.5° in embryo water containing 0.1% Methylene Blue hydrate (Sigma, St. Louis, MO, USA) and 0.03% Instant Ocean sea salt (United Pet Group, Cincinnati, OH, USA). Experiments were performed under UCSD IACUC protocol S13006.

### Hypoxia exposure and RNA isolation for microarrays

For hypoxia treatment of embryos used for RNA isolation and microarray analysis, a closed hypoxia chamber was used with a flow meter attached to a gas source (Billups-Rothenberg, Inc.). Hypoxia chambers were flushed for 4 min at 20 L/min, and repeated 30 min later with nitrogen gas. Also, 50-mm Petri dishes containing 4 ml of embryo water were pre-equilibrated in the hypoxia chamber for at least 4 hr prior to embryo transfer because this was determined to be the minimum time necessary to reach <1% oxygen as measured by a dissolved oxygen meter and gave similar viability results when compared with longer pre-equilibration times up to 24 hr (DO-5509; Alfa Electronics). A colorimetric resazurin indicator was used to monitor the hypoxic environment during pretreatment of media and the duration of each experiment (<1% oxygen if colorless; Bio-Bag, Becton Dickinson and Company).

Hypoxia was induced by transfer of individual embryos into hypoxic media at the shield or 8-somite stage for 1 to 2 hr, as indicated. Embryos from synchronized crosses were dechorionated at <1 hr post-fertilization (hpf) and maintained in a normoxic environment. Embryos were examined individually prior to hypoxia exposure and any delayed or abnormal embryos were discarded. Hypoxia exposure was initiated by individual embryo transfer from normoxic to hypoxic media using a glass pipette. Care was taken to minimize chamber opening time and gas flow was turned on immediately prior to and during chamber opening to minimize atmospheric contamination of chamber environment. After embryo addition, the resealed chamber was flushed with gas using the same protocol as for initial pre-equilibration above.

After hypoxic incubation, embryos were removed from the hypoxia chamber and immediately transferred in groups of 10–15 embryos to 1.5-ml tubes for flash-freezing in liquid nitrogen and storage at −80°, and samples were collected in duplicate. Frozen embryos were subsequently thawed on ice and pestle-homogenized in an initial 250 μL of TriZol (Life Technologies), to which 500 μL was added once homogenization was complete and the standard extraction protocol completed. RNA was quantitated using a NanoDrop and quality was verified using an Agilent BioAnalyzer prior to further processing.

### Microarray hybridization and analysis

High-quality RNA was amplified with the MessageAmp kit (Ambion) and labeled fluorescently with Cy3 or Cy5-dUTP (Amersham) in a reverse-transcription reaction using Superscript (Invitrogen). Labeled cDNA was rinsed and pooled with 20 μg of each of salmon sperm DNA, poly-A RNA, and tRNA, which were then concentrated to a small volume in a Microcon YM-30 column. Samples were added to full-genome zebrafish microarrays designed using the Zv7 genome assembly and containing 385,000 probes at approximately 12 probes for each of 37,157 genes (071105_Zv7_EXPR; NimbleGen, Inc.) (Bai *et al.* 2010). Hybridization, washes, and scanning were performed per standard protocol. Biological replicates were performed with “dye-flipping” so that each duplicate sample was labeled with the opposite dye as the first experimental sample. The resulting dataset was filtered and analyzed with R/Bioconductor and Limma to confirm normal distributions of intensities and the absence of significant region-specific artifact prior to inclusion into the dataset for normalization and downstream analysis. An established empirical Bayesian method for differential expression in microarrays (eBayes) was used to determine *P* values of individual genes taking into account multiple hypothesis testing, the overall data distribution for all genes, and the small number of biological replicates typical in genome-wide datasets (Smyth 2004, 2005). Microarray source data are available at NCBI GEO (Accession number: GSE68473; http://www.ncbi.nlm.nih.gov/geo/), and the R code used for the analysis can be reviewed in full in the Supporting Information, File S1. Top hypoxia-induced genes were filtered to retain only those genes that had a single unambiguous probe BLAT match to the later Zv9 genome assembly, for which RNA-seq or EST evidence was available (UCSC genome browser, Z-seq) (Pauli *et al.* 2012), and for which an identifiable human homolog existed in RefSeq and/or Ensembl databases (Table S1 and Table S2).

### Quantitative RT-PCR

Embryos were pestle-homogenized in 1.5-ml microfuge tubes in lysis buffer and the standard RNA extraction protocol was completed using the RNeasy kit (Qiagen). RNA was converted to cDNA using iScript (Bio-Rad), and qPCR reactions were run on an Opticon 2 Real-Time Cycler (Bio-Rad). See Table S4 for a list of primers used. Fold difference was determined with the comparative Ct method in Microsoft Excel; Δ(Ct) = (Ct, hypoxia-inducible gene) − (Ct, reference gene); ΔΔCt = Δ(Ct, non-stressed control) − Δ(Ct, hypoxia-exposed). Melting curve analysis was performed after the last amplification round to ensure the presence of a single amplified product.

We used exonic primers designed to amplify the MO-targeted exon, the predicted retained intron (if present), and at least a portion of the next downstream exon for *btr01*, *camk2g2*, *crtc3*, *ncam2*, *inhbb*, *opn5*. The MO-induced abnormal mRNAs with retained introns therefore would not be expected to amplify under the short cycle times optimized for qPCR products <250 bp in size and, furthermore, the introduction of stop codons from intronic sequence would predispose these abnormal products to nonsense-mediated decay. For *ttll11*, a region downstream of the MO-targeted splice site was amplified.

### Morpholino injection, developmental hypoxia assay, and phenotypic scoring

MOs were designed and obtained from Gene Tools, LLC. Splice-blocking MOs were preferred, but translation-blocking MOs were used if no splice-blocking MO could be designed, such as for *irs2*. MO oligonucleotides were solubilized in water at a stock concentration of 1 mM (∼8 mg/ml). The resulting stock solution was diluted 1:4 (∼2 mg/ml) in water containing a phenol red tracer. MOs used as injection controls: MO1-*dnd1* (GCTGGGCATCCATGTCTCCGACCAT), targeted to the germ cell gene *dnd1* and the standard control oligo (CCTCTTACCTCAGTTACAATTTATA). See Table S3 for h-MO sequences.

Because consistency of phenotype and survival of MO-injected embryos were observed to be higher when chorionated, we used chorionated embryos for MO microinjection and subsequent hypoxia exposure for the developmental stress assay. Wild-type embryos at the one- to four-cell stage were injected with MO into the yolk near the embryo interface at a volume of 1 to 2 nl (2–4 ng). All MO were injected at 2 ng except for *ncam2*, which was injected at 4 ng. Control MO injections were matched per experiment to h-MO amounts and volumes injected.

For mRNA injection experiments, we injected embryos into the cytoplasm at the one-cell stage with full-length mRNA. For MO rescue experiments, we subsequently re-injected separately into the yolk with a different needle containing MO at or prior to the four-cell stage. Full-length mRNA for each gene of interest was generated via PCR and directional cloning into pCS2p+ (Addgene), amplified from a single colony, and sequence-verified in entirety (Table S4 and Table S5), with the exception of *irs2*, which was obtained commercially (ATCC #10169386). For splice-blocking MOs, the standard cDNA sequence was used for mRNA rescue (*crtc3*, *brt01*). Because the *irs2 MO* was translation-blocking, we modified the cDNA region immediately downstream of the ATG corresponding to the MO binding site to introduce synonymous mutations that would retain amino acid identity but not be bound by the MO. Accordingly, the initial sequence of ATG-GCA-AGT-CCG-CCT-CTT-AAA-GGG-G was modified to ATG-GC**G-**AG**C**-CC**A**-CC**G**-CT**G**-AA**G**-GG**C-**G (changed bases in bold), both of which translate to the peptide MASPLKG (QuikChange Lightning Site Directed Mutagenesis Kit; Agilent). Plasmids were linearized with the appropriate restriction enzyme and then transcribed with SP6 RNA polymerase to generate full-length mRNAs for rescue/overexpression experiments (Ambion). RNA was quantitated with a Nanodrop (Thermo Fisher Scientific, Inc.). After injection, embryos were viewed at 1-hr and 4-hr timepoints and discarded if they showed asymmetric dye uptake, delay, or significant aberrations.

For hypoxia treatment of morphant embryos, an adjustable, self-regulating hypoxia chamber was used at 0.3% oxygen in a 28.5° humidified incubator (Biospherix C-chamber). Embryos were individually transferred with a pipette into 4 ml of pre-equilibrated hypoxic media in 6-well plates for hPC for 5 hr at 1 d post-fertilization (dpf) and/or prolonged sH for 38 hr at 1.5 dpf, both at 0.3% oxygen. To end hypoxia exposure, plates were removed from the hypoxia chamber, 2 ml of normoxic embryo water was added to each well, and wells were kept open to the ambient air for 10 min prior to replacement of lids and return to the normoxic 28.5° incubator. During normoxic recovery, embryos were checked daily for lethality and were provided fresh water.

Phenotypes were scored at 5 dpf or the approximate developmental equivalent if developmentally delayed by prolonged sH, with samples exposed to 38 hr of 0.3% oxygen scored for phenotype at 6–7 dpf to adjust for the approximate 1.5 d of delay. Phenotypic scoring was performed as follows: mild—small or absent swim bladder, fin shape or texture abnormalities, fin hyperpigmentation, diminished touch response, or asymmetric resting postural tone; severe—body axis deformity, pericardial or generalized edema, irregular somites, absent fins, absent touch response, failure to hatch, hemorrhage, single or small eyes, or necrotic/opaque tissue; and dead—widespread/brain necrosis or absence of heartbeat. If a single fish contained both mild and severe category abnormalities, then the fish was scored as severely abnormal.

### *In situ* expression analysis

Digoxigenin (DIG)-labeled antisense RNA probes to desired gene transcripts were *in vitro* transcribed from linearized plasmid templates containing the clone of interest with T7 or SP6 RNA polymerase as appropriate (Promega). Primers used to amplify and clone probe regions are listed in Table S4. The *in situ* hybridization of zebrafish embryos was performed according to standard procedures (Thisse and Thisse 2008) using immunohistochemical detection of the DIG-labeled RNA–RNA hybrids by an anti-DIG alkaline-phosphatase coupled antibody and nonfluorescent, colorimetric detection with BCIP/NBT. Digital images were obtained on a Zeiss Axio-Imager microscope and scaled, cropped, and contrast-adjusted with Photoshop.

#### Statistics and general methods:

For embryo injection and/or stress experiments, embryos were examined immediately prior to initiation of stress and were excluded if they exhibited developmental delay and/or malformations. This criterion was pre-established. Embryos with normal development and morphology were then assigned randomly to control or experimental protocols, and the order of injection of control and experimental embryos was alternated for successive experiments. After exposure to stress, embryo samples were labeled numerically and survival from each well was quantitated prior to assignment of identity. Sample sizes were constrained by hypoxia chamber size and embryo density limits as follows: each hPC experiment was performed with four groups to include relevant controls—injection alone, hPC alone, sH alone, and hPC+sH; a maximum of 32 separate biological samples was possible per experiment using two hypoxia chambers, each of which held four separate four-well plates; and a total of 32 samples for four treatment groups yields eight different biological samples, or four treatments with biological duplicates (one control plus three experimental treatments). Each well contained a minimum of eight and a maximum of 12 embryos to prevent crowding, which can cause abnormal, delayed, and/or asynchronous embryos. Biological duplicates were placed on opposite corners of plates to minimize edge or location effects in the hypoxia chamber. Statistical comparisons for embryo survival experiments were made with a *t*-test (unpaired, one-tail) as different samples were compared, and in each case the inhibition or overexpression of a gene product was expected to decrease or increase hypoxia tolerance, respectively. Variance is indicated using SEM and sample size varied across all data from n = 2 to n = 8, and is indicated for each specific panel in the figure legend. Please see *Microarray hybridization and analysis* for discussion of statistical methods used for genome-wide data.

## Results

### Acute hypoxic stress response in the embryonic zebrafish

To establish the tolerance of the developing zebrafish to acute hypoxia, we placed individual embryos into pre-equilibrated hypoxic media at specific developmental stages for different lengths of time. After hypoxic exposure, embryos were returned to normoxic media and scored for viability and phenotype at 5 dpf. Survival to hypoxia exposure varied with developmental time of onset, length of exposure time, and severity of hypoxia (Figure S1). We found that exposure to 0.3% oxygen induced consistent, strong lethality when compared with 1% oxygen exposure; therefore, we selected 0.3% oxygen for our severe hypoxia (sH) exposure experiments. Consistent with other reports, we found that older larvae were less hypoxia-tolerant than younger embryos and larvae (Mendelsohn *et al.* 2008; Tucker *et al.* 2011). We observed developmental arrest in our 0.3% oxygen-exposed embryos, a phenomenon that was described previously for complete anoxia in zebrafish and *Drosophila* embryos (Douglas *et al.* 2001; Mendelsohn *et al.* 2008; Padilla and Roth 2001). The hypoxia-induced arrest we observed was reversible, because embryos exposed to 0.3% oxygen at 0.5 dpf (bud stage) remained developmentally arrested at or near bud stage after 12 hr of sH, but restarted development normally after return to normoxia and remained 100% viable (Figure S2). After recovery, larvae were morphologically indistinguishable from controls, except for an approximate 12-hr delay in development, corresponding to time spent arrested in 0.3% oxygen. However, prolonged sH exposure during embryogenesis resulted in phenotypic defects at all stages tested, including pericardial edema, generalized edema, and occasional hemorrhage.

### Hypoxic preconditioning is protective against acute hypoxia in the embryonic zebrafish

**Figure 1:**
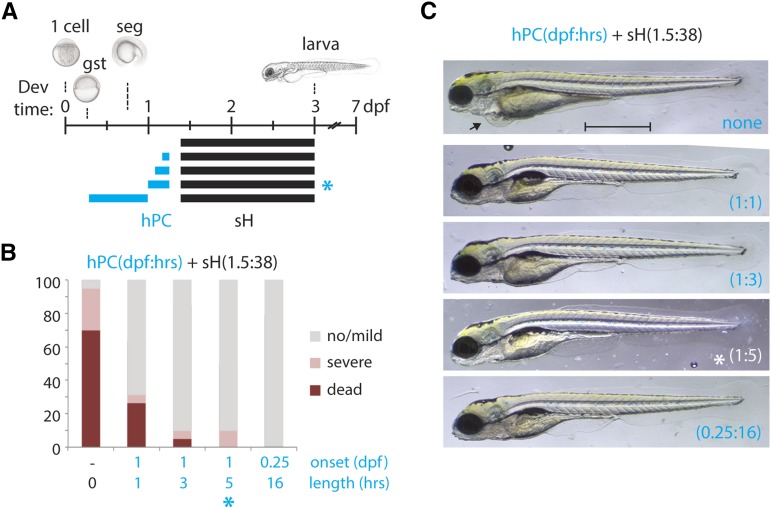
Hypoxic preconditioning can protect against acute hypoxia-induced injury. (A) Schematic indicating developmental exposure at 1.5 dpf to 0.3% oxygen for 38 hr (sH; black bars) with various prior hypoxic preconditioning regimens (hPC; blue bars) of 0.3% oxygen at 1 dpf for 1, 3, or 5 hr, or at 0.25 dpf for 16 hr followed by 4 hr of normoxic recovery prior to sH. The asterisks indicate the optimal hPC protocol used for subsequent studies. (B) Percentage distribution of phenotypes from each hPC protocol as shown in (A), grouped by severity of defects at 5 dpf. hPC larvae are highly protected against sH relative to naïve controls, >95% of which die or develop severe malformations. (C) Representative surviving larvae from each hPC protocol as in (A). Note the pericardial edema in a nonpreconditioned survivor of sH (arrow). Scale bar = 1 mm. n = 2–4 biological replicates per condition for (B). gst, gastrula; seg, segmentation.

Because hPC had been demonstrated in adult zebrafish (Rees *et al.* 2001), we sought to establish conditions under which it would also occur during early development. We were able to induce hPC in zebrafish embryos by a brief incubation in 0.3% oxygen, followed by a period of recovery prior to a prolonged re-exposure to 0.3% oxygen. Figure 1, A–C indicates several different parameters tested for hPC, from which we determined the most optimal effect of hPC to be 5 hr of sH at 1 dpf and 4 hr of normoxic recovery prior to re-exposure to sH for 38 hr at 1.5 dpf: sH(1d:5h + 1.5d:38h) (blue asterisk; *Materials and Methods*). This regimen maximized the differential lethality of the sH(1.5d:38h) protocol between naïve and hPC embryos (70% *vs.* 0%; Figure 1B). The protective effect of hPC was dose-dependent because preconditioning for 1 or 3 hr gave a lesser survival advantage compared to 5 hr or more of hPC, which was almost completely protective against sH (Figure 1B). Preconditioned animals had increased survival and milder phenotypes than their naïve counterparts, the overwhelming majority of which suffered death or major deformation (Figure 1, B and C).

### Whole-genome transcriptional profiling of the acute hypoxia response

**Figure 2:**
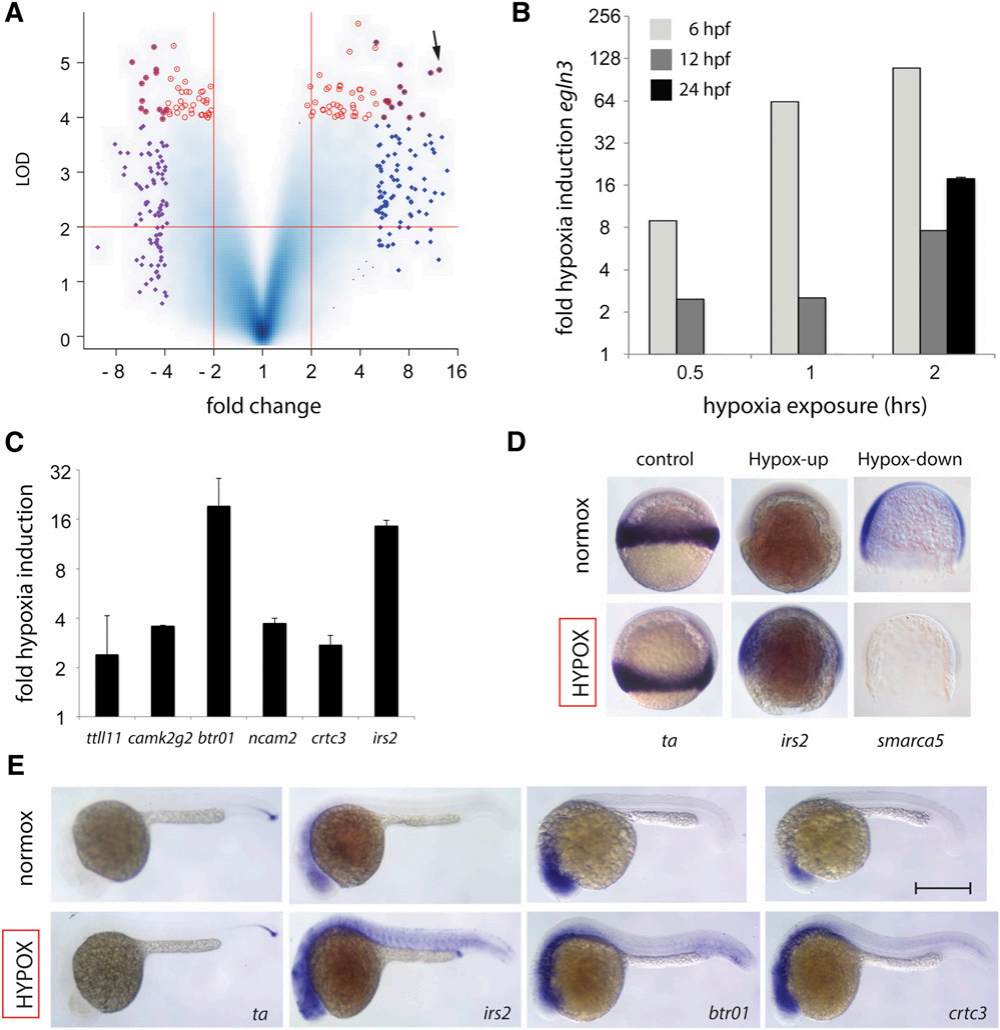
Genome-wide expression screen identifies novel hypoxia-regulated genes. (A) Log-log volcano scatterplot depicting differential expression and statistical significance of hypoxia-regulated genes during zebrafish development. LOD (log_10_ odds) score is on the y-axis and log_2_ differential expression is on the x-axis, with two-fold increase, two-fold decrease, and *P* = 0.01 significance thresholds demarcated by red lines. The plot represents 26,259 genes that showed significant expression in all hybridization experiments performed, with higher blue intensity used in confluent areas to indicate increased density of multiple overlapping individual genes. The 100 most differentially upregulated and downregulated genes are highlighted with blue and purple diamonds, respectively, and red circles indicate the 100 genes with the highest statistical significance scores. *egln3* is indicated as a highly induced, highly significant gene (arrow). (B) *egln3* shows dose-dependent hypoxia induction at 6 hpf (shield) and 12 hpf (8-somite) to 0.5 hr, 1 hr, and 2 hr of 0.3% oxygen exposure, and is also hypoxia-induced at 24 hpf. Relative qPCR induction to housekeeping control gene *eef1a1l1* is shown. (C) Validation of additional hypoxia-induced genes via qRT-PCR. (D) *In situ* hybridization at gastrula (shield) of representative hypoxia-upregulated (*irs2*), and hypoxia-downregulated (*smarca5*) genes as compared to control gene *ta*, a zebrafish *brachyury* homolog unchanged by hypoxia. (E) *In situ* hybridization at late segmentation/early pharyngula (1 dpf) of hypoxia upregulated genes *irs2*, *btr01*, and *crtc3* as compared with control gene *ta*. Scale bar = 0.5 mm; n = 2 biological replicates per condition for (B) with SEM (C), except that the 6 hpf and 12 hpf samples from (B) are the average of two technical replicates and error bars are therefore omitted.

Next, we hypothesized that genes required either for protection against acute hypoxia or for establishment of the hPC protective state are likely to be activated by the initial, brief hypoxia exposure. We set out to identify these hypoxia-activated genes by comparative analysis of differential RNA expression between embryos exposed to acute hypoxia and their normoxic controls. We further hypothesized that genes whose differential activation by hypoxia is conserved across different developmental stages in zebrafish are more likely to be components of a core hypoxia response pathway conserved to humans. For both of these reasons, we utilized genome-wide transcriptional profiling at two different stages of development: gastrula (shield) and segmentation (8-somite). Full-genome zebrafish expression microarrays representing 37,157 unique zebrafish genes were used to measure global transcript levels under control and hypoxia exposure (0.3% oxygen for 2 hr) in duplicate at the two developmental stages (Figure 2, Figure S3). A summary volcano plot of the 26,259 genes for which signal was measured shows the range of differential expression between control and hypoxic stress experiments, with multiple genes passing more than two-fold change and *P* < 0.01 significance criteria (Figure 2A). Analysis of these data prominently identified the prolyl hydroxylase *egln3*, a key regulatory enzyme of the Hif1a response pathway (Kaelin and Ratcliffe 2008; Santhakumar *et al.* 2012). *egln3* was among the most highly upregulated and statistically significant genes detected under hypoxic stress at 6 hpf (gastrulation, >100-fold), 12 hpf, and 24 hpf (segmentation, >10-fold) (Figure 2, A and B). Multiple additional novel or uncharacterized hypoxia-regulated genes were identified via differential expression or correlational clustering with *egln3* (Table S1, Table S2, and Table S6). Hypoxia inducibility of *egln3* and other hypoxia-upregulated genes were validated by quantitative RT-PCR (qPCR), including *irs2*, *btr01*, and *crtc3* (Figure 2, B and C). *In situ* hybridization further verified the upregulation of *irs2*, *btr01*, *crct3*, and *egln3* and the downregulation of *basigin*, *smarca5*, and *ythdf2* after hypoxia exposure at gastrula and/or segmentation stages when compared to controls (Figure 2, D and E and Figure S3).

### *irs2* is a novel acute hypoxia-protective gene

**Figure 3:**
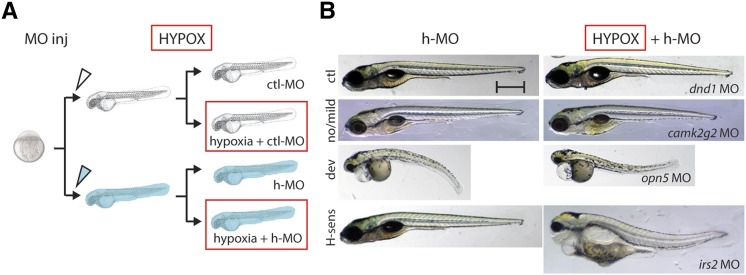
Knockdown of hypoxia-induced stress candidate genes identifies a stress-sensitive phenotypic class. (A) Outline of functional acute hypoxia screen performed on morphants of the 10 candidates validated from the initial differential expression screen as described. Embryo microinjection was performed for each MO, after which control-injected morphant (ctl-MO; clear) and hypoxia target morphant (h-MO; blue) larvae were then divided into control and hypoxia-exposure (red box) groups, resulting in four different groups in total—ctl-MO injection only, ctl-MO + hypoxia, h-MO injection only, and h-MO + hypoxia. (B) Different phenotypic classes from a moderate hypoxic stress protocol sH(1.5d:30h) for the 10 genes tested by MO knockdown are shown: “no/mild” with no defects or minor defects only in the fin or air bladder **(**five genes, *camk2g2* shown); “dev” have severe defects such as curved body axis or pericardial edema irrespective of hypoxia treatment (four genes, *opsin5* shown); and “hypoxia-sensitive,” where morphant phenotype is greatly exacerbated under hypoxia (one gene, *irs2*). *dnd1*, whose expression and function are restricted to the developing gonad, is used as a control MO. See *Materials and Methods* for detailed phenotypic scoring criteria. Scale bar = 0.5 mm.

Hypoxia-activated genes identified by microarray at gastrula and segmentation stages were further validated by qPCR and/or *in situ* hybridization (Figure 3). Genes demonstrating hypoxia-inducibility at 1 dpf, and therefore conservation of response across several phases of development, were preferentially chosen for MO knockdown in a secondary functional acute hypoxia sensitivity screen (Table 1, Figure 3). The rationale for selection of hypoxia-upregulated genes was that MO knockdown could uncover a necessary and protective role, therefore resulting in decreased resistance against sH. In contrast, the prediction and interpretation of results from knockdown of hypoxia-repressed genes were anticipated to be less straightforward and are not a focus of this study.

**Table 1:** Hypoxia candidate gene qPCR validation at 1 dpf*^a^*

Gene	1 dpf Hypoxia Inducibility (log_2_)	SEM
***btr01***	4.3	0.6
***irs2***	3.9	0.1
***gabra5***	2.1	0.7
***ncam2***	1.9	0.1
***camk2g2***	1.8	0.0
***crtc3***	1.5	0.2
***ttll11***	1.3	0.8
*cdk19*	0.9	0.1
*grip2*	0.7	0.5
*rbms2*	0.7	0.1
*hrg*	0.6	0.2
*nbr1*	0.6	0.1
*gabrr3*	0.5	0.2
*c6orf191*	0.5	0.3
*ankar*	0.5	0.3
*erc1b*	0.3	0.2
***inhbb***	0.3	0.3
*sox5*	0.3	0.3
*ldb1a*	0.2	0.2
*atp2a1*	0.2	0.1
*epn3*	0.2	0.4
*ngf*	0.1	0.1
*rfp138*	0.0	1.7
*trpm6*	−0.1	0.1
*c6orf94*	−0.3	0.7
*pde4c*	−0.7	0.3
*cox18*	−0.7	0.0
***opn5***	−0.8	0.6
***mical2***	−1.4	0.1
*msrb3*	−1.4	0.4
***pacsin3***	−1.6	0.2
*serp2*	−2.3	1.1

a Genes whose morphants were tested in the developmental hypoxia stress assay are indicated in bold.

**Figure 4:**
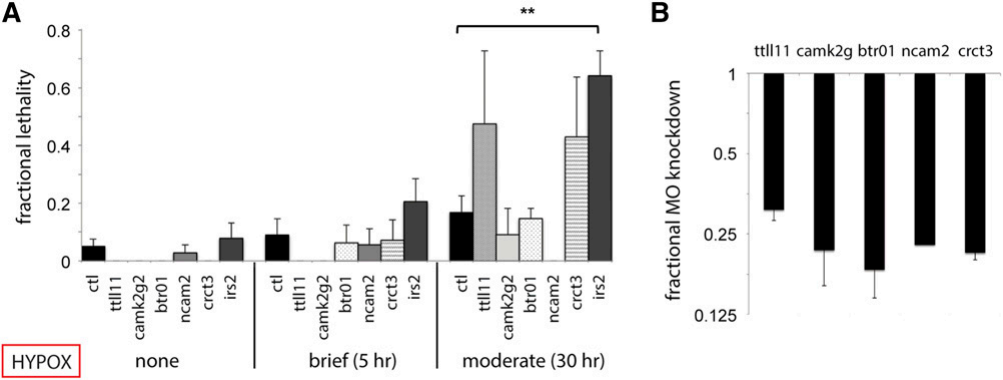
*irs2* is required for acute hypoxic stress buffering. (A) h-MO knockdown survival is shown in comparison to ctl-MO survival under the following hypoxia exposures: none (MO injection alone); brief sH(1d:5h); and moderate sH(1.5d:30h). *irs2* inhibition significantly impairs moderate sH survival (*P* = 0.0029). (B) qRT-PCR validation of targeted splice h-MO knockdown is shown for *ttll11*, *camk2g2*, *btr01*, *ncam2*, and *crct3*. Note that only a translation-blocking MO was obtainable for *irs2* because it only contains a single intron that is unsuitable for MO targeting; therefore, qPCR was not performed. n = 2 to 8 biological replicates with SEM for (A) and (B).

Accordingly, antisense MO oligonucleotides were designed and tested against 10 representative hypoxia-responsive genes among those with the highest differential expression shared between gastrulation and segmentation embryos and those with highest *egln3* correlation (Figure 3, Figure 4, Table 1). Morphant embryos for these candidate hypoxia-protective genes were exposed to both brief and moderate hypoxia protocols, sH(1d:5h) and sH(1.5d:30h), because these protocols caused low phenotypic injury in controls and were therefore poised to detect exacerbations of lethality due to MO inhibition of hypoxia-protective gene function (<10% and <20%, respectively; Figure 3B, Figure 4A). At least 100 embryos were injected per MO for each gene analyzed. Knockdown of the intended MO target for splice-blocking MOs was validated by qPCR, because the predicted aberrant splice product with a retained intron is often too large to amplify or more rapidly degraded due to nonsense-mediated decay (Bill *et al.* 2009; Draper *et al.* 2001; Knapp *et al.* 2006). Accordingly, targeted MO knockdown was found to decrease gene expression levels down to ∼15–30% of normal (Figure 4B).

MO-injected embryos were subsequently classified into three phenotypic groups based on their phenotypes in normoxia and hypoxia: none/mild defects, developmental defects, and hypoxia-sensitive (Figure 3B). Of the 10 hypoxia-induced genes tested via MO knockdown, a single gene, *irs2*, demonstrated an acute hypoxia-sensitive phenotype. *irs2* morphants showed normal/mild phenotypes under normoxia but exhibited exaggerated malformations and lethality under moderate hypoxic stress conditions (*P* = 0.0029; Figure 4A). MO targeting of four other genes resulted in developmental phenotypic defects independent of hypoxia treatment, *inhbb*, *opn5*, *mical2b*, and *pacsin3*, whereas five genes showed no significant difference in survival from control MO-injected embryos: *ttll11*, *camk2g2*, *btr01*, *ncam2*, and *crtc3*. The defects involving multiple tissues under both normoxia and hypoxia are likely due to a requirement of these genes for normal development, such as for *pacsin3* and notochord development (Edeling *et al.* 2009). The hypoxia-protective function of genes whose knockdown caused developmental defects cannot be ruled out, but these genes were not pursued further in this study.

### Hypoxic preconditioning requires *irs2*, *crct3*, *btr01*, *camk2g2*, and *ncam2*

**Figure 5:**
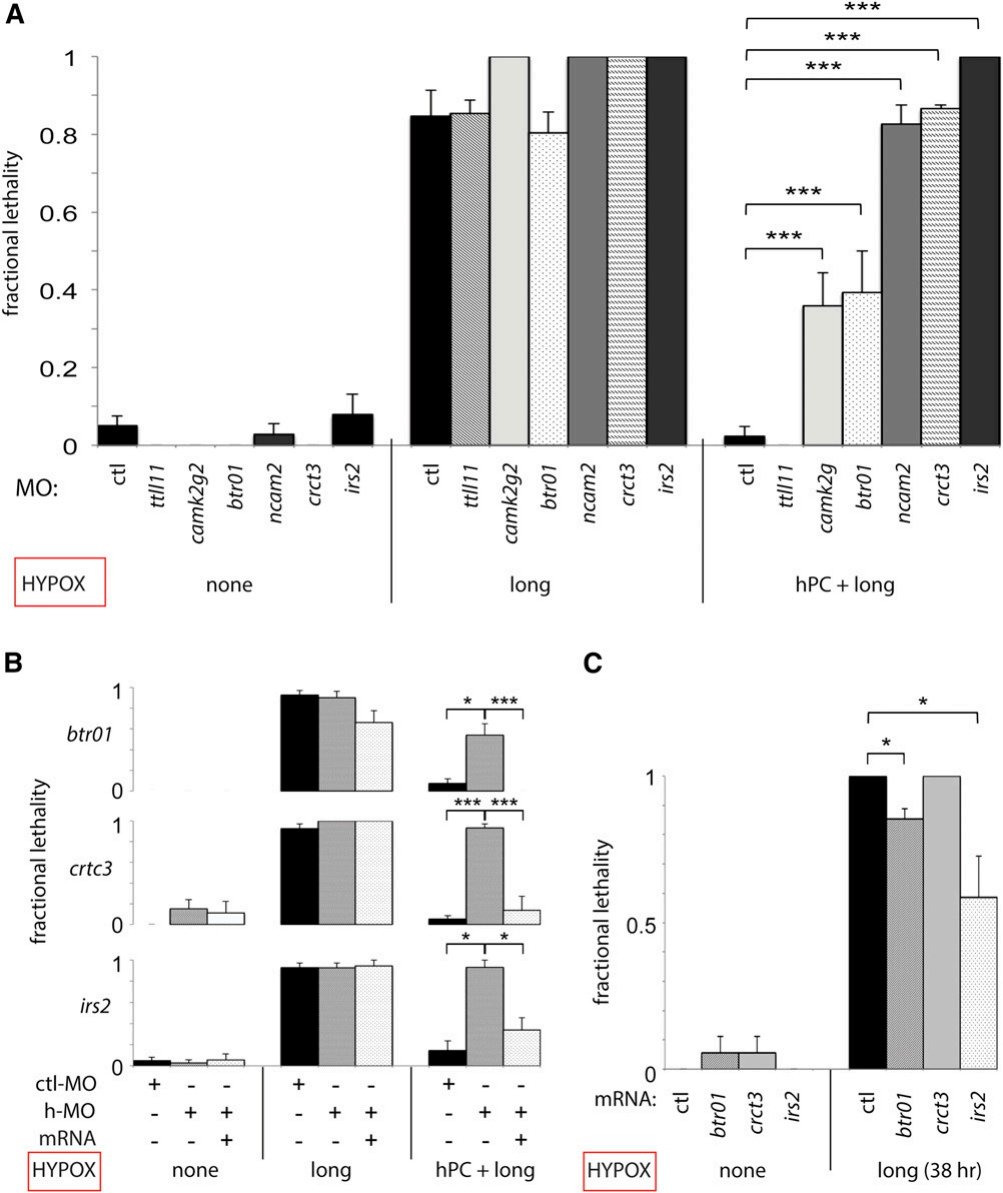
Multiple acute hypoxia response genes are required for the protective effect of hypoxic preconditioning. Hypoxia survival graphs with fractional lethality at 24 hr are shown on the y-axis. (A) h-MO knockdown of candidate hypoxia-protective genes is shown in comparison to ctl-MO in untreated controls (injection alone), prolonged sH(1.5:38), and hPC + prolonged sH embryos. Impaired hPC is demonstrated at *P* < 0.0001 in the following morphants: *camk2g2* (*P* = 6.7 × 10^−4^); *btr01* (*P* = 7.3 × 10^−4^); *ncam2* (*P* = 1.7 × 10^−6^); *crtc3* (*P* = 6.2 × 10^−7^); *and irs2* (*P* *=* 2.6 × 10^−7^); but not in *ttll11*. (B) h-MO knockdown inhibits hPC and is rescued by mRNA coinjection (*btr01*, *P* = 0.0038, 0.015; *crct3*, *P* = 1.0 × 10^−6^, 7.2 × 10^−4^; *irs2*, *P* = 0.033, 0.049*)*. (C) Hypoxia response gene overexpression via mRNA injection is sufficient to confer partial protection from prolonged hypoxia [*btr01*, *P* = 0.027; *irs2*, *P* = 0.050; sH(1.5d:38h) protocol]; n = 2 to 8 biological replicates with SEM for (A–C).

Hypoxia-activated genes identified by microarrays and validated by qPCR and/or *in situ* hybridization were then tested using the optimized preconditioning protocol sH(1d:5h + 1.5d:38h), depicted in Figure 1. Of the six morphants without developmental defects tested, five showed significant impairment of hPC in comparison to control MO–injected embryos: *irs2*, *crct3*, *btr01*, *camk2g2*, and *ncam2* (Figure 5A). For *btr01*, *crct3*, and *irs2*, we additionally demonstrated that the loss of protective hPC in morphants could be restored by full-length mRNA rescue (Figure 5B).

### Overexpression of *irs2* and *btr01* protects against acute hypoxic stress in the absence of preconditioning

During MO knockdown and rescue experiments, we noted that *btr01* morphants injected with rescue *btr01* mRNA showed a trend toward lower lethality when subjected to sH in the absence of hPC (Figure 5B). Although this finding did not meet strict significance criteria (*P* = 0.056), we were intrigued regarding whether any genes required for hPC could confer hPC-like protection against hypoxia via overexpression alone. We tested this with mRNA injection followed by exposure to protocol sH(1.5d:38h) without hPC, and we found that embryos with *irs2* or *btr01* overexpression showed improved survival. This result suggests that activation of either of these two genes is partially sufficient to provide the protective effect of hPC, whereas *crtc3* overexpression is not (Figure 5C). Overexpression or MO knockdown alone did not cause major phenotypic abnormalities or developmental delay (Figure S4).

## Discussion

The molecular mechanism of hPC remains incompletely characterized despite considerable previous literature, and both HIF and non-HIF-mediated mechanisms have been postulated (Gidday 2006; Semenza 2014). Our approach to identify relevant hypoxia-response genes was based on global differential expression and *in vivo* functional validation, and we believe this strategy to be neutral with regard to pathway and, therefore, equally likely to uncover both HIF-mediated and non-HIF-mediated factors. We identified *irs2*, *crtc3*, *camk2g2*, *btr01*, and *ncam2* as hypoxia-induced genes whose morphants exhibited impaired or absent hPC (Figure 5). Of these, only *irs2* is a known HIF pathway target (Taniguchi *et al.* 2013; Wei *et al.* 2013). We previously observed dramatic upregulation of the HIF target gene *egln3* at 6, 12, and 24 hpf (Figure 2B). Several lines of evidence suggest that the HIF pathway is not active and/or contributes little to hypoxia response or protection at this early time in development (Mendelsohn and Gitlin 2008; Robertson *et al.* 2014). Therefore, the early hypoxia-inducibility of *irs2* and *egln3* is likely due to non-HIF transcriptional activators.

Notably, *irs2*, *crtc3*, and *camk2g2* are all known to function in glucose and/or lipid metabolism in other systems, but none of the zebrafish genes have been studied to date. The mouse *irs2* has been shown to modulate cell growth and metabolism downstream of insulin receptor signaling, and irs2^−/−^ knockout mice develop type 2 diabetes (Long *et al.* 2011; Withers *et al.* 1998). A link between insulin pathway signaling and hypoxia survival has been reported in model organism studies in *C. elegans*, *Drosophila*, and most recently in mouse (Dekanty *et al.* 2005; Scott *et al.* 2002; Taniguchi *et al.* 2013; Wei *et al.* 2013). In our study, we found *irs2* to be prominently upregulated and to be ectopically activated in multiple tissues in response to hypoxia (Figure 2). *irs2* morphants showed decreased survival after acute sH and were also deficient in hPC (Figure 3, Figure 4, and Figure 5).

*crtc3* is a transcription factor downstream of cAMP and CREB signaling and, like *irs2*, it has been implicated in energy metabolism. In mice, it is predominantly expressed in adipose tissue, and mice lacking *crtc3* are obesity-resistant when fed a high-fat diet, with smaller adipocytes and higher baseline lipolysis (Song *et al.* 2010). In humans, a specific *crtc3* polymorphism is associated with obesity. We provide the first functional characterization of zebrafish *crtc3* via its hypoxia inducibility and requirement for hPC.

*camk2g2* is one of six different zebrafish *camk* homologs transcriptionally active during zebrafish embryogenesis and is located on chromosome 13 (Rothschild *et al.* 2007). Several of the *camk* homologs were shown to function in left–right asymmetry in zebrafish. However, *camk2g2* was unlike others in that it was not expressed near the Kupffer’s vesicle, nor did its knockdown result in laterality defects, suggesting a different function (Francescatto *et al.* 2010). Mouse *camk2g2* knockouts also show improved insulin sensitivity and lower hepatic glucose production, consistent with the direction of effect of *irs2* and *crtc3* on glucose metabolism (Ozcan *et al.* 2013). In addition, the developmental expression of *camk2g2* is very similar to that of *irs2* and *crtc3* in zebrafish and provides further evidence for a combined role of these genes in buffering hypoxic stress in the same tissues (Figure 2, D and E) (Francescatto *et al.* 2010).

Furthermore, several of these acute hypoxia response genes are implicated in human pathological states of metabolic imbalance. Loss-of-function mouse mutants in *irs2*, for example, develop type II diabetes (Long *et al.* 2011), and specific polymorphisms in human *irs2* are associated with decreased or increased risk for development of type II diabetes (Lautier *et al.* 2003; Mammarella *et al.* 2000). Interestingly, hypoxia-induced *irs2* activation was recently shown to be mediated in the human liver via *Hif2a*, which has itself been separately implicated in hypoxic preconditioning in mouse skeletal muscle and other tissues (Taniguchi *et al.* 2013; Wei *et al.* 2013). In the case of *crtc3*, humans with gain-of-function polymorphisms are at higher risk for obesity, and homozygous *ctrc3*-deficient mutant mice are resistant to obesity even when fed a high-fat diet (Qi *et al.* 2009; Song *et al.* 2010). *crtc3* and *irs2* therefore appear to be “thrifty” genes that enhance survival under starvation conditions. We suggest this interpretation can be broadened to include an advantageous role in energy parsimony for *crtc3* and *irs2* in protection from hypoxia, which is another state of low energy availability.

The hypoxia-induced developmental arrest in zebrafish embryos we observed is similar to that previously described for anoxia and metabolic inhibition (Mendelsohn and Gitlin 2008; Mendelsohn *et al.* 2008; Padilla and Roth 2001). It is of further interest to examine whether multiple low-energy states—*i.e.*, hypoxia, anoxia, and metabolic inhibition—can activate hPC. Because these perturbations can be elicited through both environmental and chemical means, one can also perform them in combination—for example, hypoxia and metabolic inhibition together—to determine whether their effects are additive and therefore suggestive of different mechanisms of action.

Our data support the hypothesis that hPC-induced hypoxia resistance is substantially due to the switching of metabolism to a low-energy use state, even at a time of relative oxygen abundance. Interestingly, this is a well-known characteristic of cancer cells addressed in the Warburg hypothesis, where glycolysis is favored over oxidative phosphorylation despite oxygen availability (Warburg 1956). Future comparative studies of the activation of these novel hPC genes between cancer cells and normal tissue will provide additional insight into whether the hPC pathway contributes to transformation and malignancy by providing a survival advantage for rapidly growing cancer cells that may outstrip their blood supply.

We have shown that prior hypoxia exposure is required to activate the protective response of hPC, but chronic or chronic-intermittent hypoxia is also associated with states of metabolic imbalance such as sleep apnea (Prabhakar and Semenza 2012). It is tempting to postulate that these metabolic imbalances result from pathologic, long-term activation of acute hypoxia response genes such as *irs2*. This conversion of a short-term adaptive response into a chronic deleterious one is analogous to stress-induced epinephrine release, which acutely triggers the protective “fight or flight” response. Although beneficial in the short-term, persistent catecholamine elevation is abnormal and damaging to the heart and other organs if chronically activated (Brotman *et al.* 2007; Dunser and Hasibeder 2009).

*btr01* and *ncam2* are novel, uncharacterized genes required for hPC. *btr01* is part of a large family of zebrafish “*bloodthirsty (bty)*-like TRIMs”, which contains 33 uncharacterized paralogs of the mammalian *TRIM39* gene (Boudinot *et al.* 2011; van der Aa *et al.* 2009). They are named after *bty*, which was shown to have a key early role in erythropoiesis and is the true zebrafish ortholog of mammalian *TRIM39* (Yergeau *et al.* 2005). TRIM proteins are E3 ubiquitin ligases with functions in cell growth, differentiation, and apoptosis (Meroni and Diez-Roux 2005). We present the first functional characterization of a member of this zebrafish *bty*-like gene family through demonstration of hypoxia inducibility and requirement for hPC (Figure 2, C–E, Figure 4). *ncam2* is one of three *ncam* genes in zebrafish, all of which are transmembrane molecules with five Ig-like domains and two fibronectin type II–like extracellular domains. It is normally expressed in a subset of hindbrain cells during zebrafish development and was of unknown function in both fish and mammals prior to this study (Mizuno *et al.* 2001).

Four hypoxia-inducible genes exhibited developmental defects on MO knockdown and were not pursued further in this study (*inhbb*, *opsin5*, *mical2*, and *pacsin3)*, but they may nonetheless play important roles in hypoxia tolerance. This type of dual function in normal development and in stress states is illustrated by the example of *Hif1a* itself, which is required early in development for vasculogenesis and also is a key mediator of the protective response to acute hypoxia. The complete *Hif1a* knockout is early embryonic lethal in mouse, and it was not until tissue-specific or conditional alleles circumvented this early lethality and allowed validation of *Hif1a* in hypoxic stress conditions *in vivo* in mammals (Iyer *et al.* 1998; Kotch *et al.* 1999). Therefore, a similar approach with conditional mutant alleles and/or titrations of MO knockdown may be necessary to elucidate a role in hypoxia buffering for these genes with developmentally defective morphants. Genes that did not demonstrate a morpholino knockdown phenotype may have been rescued by maternally contributed mRNA and/or protein, or by incomplete inhibition of zygotic transcription from the locus. The function of these genes could be more robustly inhibited in future studies via generation of null mutant alleles with absent zygotic transcription, and even further inhibited by elimination of potential maternally contributed mRNA and protein via generation of maternal-zygotic mutants (Ciruna *et al.* 2002).

Finally, our use of overall survival as an endpoint for hypoxic injury in this zebrafish study was beneficial for experimental throughput, but the organ-specific cause of lethality remains undetermined. Expansion of this initial view to include organ-specific analyses of hypoxic injury in wild-type and genetic mutant and/or MO knockdown animals could help determine the cause of hypoxia-induced death in this zebrafish developmental model. A focus on hypoxia-sensitive organs such as the heart and brain would be especially interesting.

In summary, our functional genomic studies of hypoxic preconditioning in this novel zebrafish model provide further insight into molecular mechanisms of hPC and into the relationship of hPC with known mediators of metabolism. Our identification of key genes and pathways known from prior hypoxic stress studies provides further validation of the strength and generalizability of our findings. We therefore believe that this embryonic zebrafish hypoxia model is of significant utility as a developmental genetic model for the study of hypoxia and hypoxic preconditioning. Future studies examining gene expression differences between naïve and preconditioned larvae at additional timepoints, such as after recovery from preconditioning and/or from severe hypoxia, are likely to further our mechanistic understanding of hypoxic preconditioning. Furthermore, it may be possible to extend this developmental genetic and genomic approach in zebrafish to identify novel stress-protective genes for other stress conditions, such as temperature or osmotic stress.

## Supplementary Material

Supporting Information
