# Supplementary material for: Novel Genes Critical for Hypoxic Preconditioning in Zebrafish Are Regulators of Insulin and Glucose Metabolism
Source: G3 (Bethesda). 2015 Apr 3;5(6):1107–16. doi: 10.1534/g3.115.018010 (PMC4478541; doi:10.1534/g3.115.018010)
Supplement: Supporting Information [file supp_5_6_1107__index.html]

Novel Genes Critical for Hypoxic Preconditioning in Zebrafish Are Regulators of Insulin and Glucose Metabolism — Supporting Information 

# Novel Genes Critical for Hypoxic Preconditioning in Zebrafish Are Regulators of Insulin and Glucose Metabolism

## Supporting Information for Manchenkov *et al.*, 2015

**Files in this Data Supplement:**

- Supporting Information - Figures S1-S4, Tables S1-S6, and File S1 (PDF, 471 KB)
- Figure S1 - Hypoxia tolerance varies with developmental time and severity of stress. (PDF, 170 KB)
- Figure S2 - Embryonic and larval hypoxia exposure causes reversible developmental arrest and can cause diverse developmental defects. (PDF, 203 KB)
- Figure S3 - Validation of genome-wide hypoxia transcriptome screen. (PDF, 178 KB)
- Figure S4 - Identified hypoxia target genes *irs2*, *crtc3*, and *btr01* do not show knockdown or overexpression phenotypes under normoxia. (PDF, 200 KB)
- Table S1 - Top 100 hypoxia-induced transcripts. (PDF, 123 KB)
- Table S2 - Top 100 hypoxia-repressed transcripts. (PDF, 124 KB)
- Table S3 - Morpholinos used. (PDF, 116 KB)
- Table S4 - Oligonucleotide primers used. (PDF, 119 KB)
- Table S5 - Full-length cDNA sequence information. (PDF, 145 KB)
- Table S6 - Genes with expression most highly correlated to *egln3* under all conditions, n = 6. (PDF, 130 KB)
- File S1 - R/Bioconductor code for bioinformatic analyses. (.txt, 7 KB)
