# Supplementary material for: Novel Genes Critical for Hypoxic Preconditioning in Zebrafish Are Regulators of Insulin and Glucose Metabolism
Source: G3 (Bethesda). 2015 Apr 3;5(6):1107–16. doi: 10.1534/g3.115.018010 (PMC4478541; doi:10.1534/g3.115.018010)
Supplement: Supporting Information [file supp_g3.115.018010_018010SI.pdf]

G3·Genes | Genomes | Genetics

Novel genes critical for hypoxic preconditioning in zebrafish are regulators of insulin and glucose metabolism

T. Manchenkov[*], M. P. Pasillas[*], G. G. Haddad[§,†], and F. B. Imam[*,†]

Department of Pediatrics (Divisions of [*]Neonatology and [§]Respiratory Medicine), University of California San Diego School of Medicine, La Jolla, California, and [†]Rady Children's Hospital, San Diego, United States of America

Correspondence should be addressed to:

9500 Gilman Drive #0734

La Jolla, CA 92093-0734

(858) 822-4661

f2imam@ucsd.edu

DOI: 10.1534/g3.115.018010

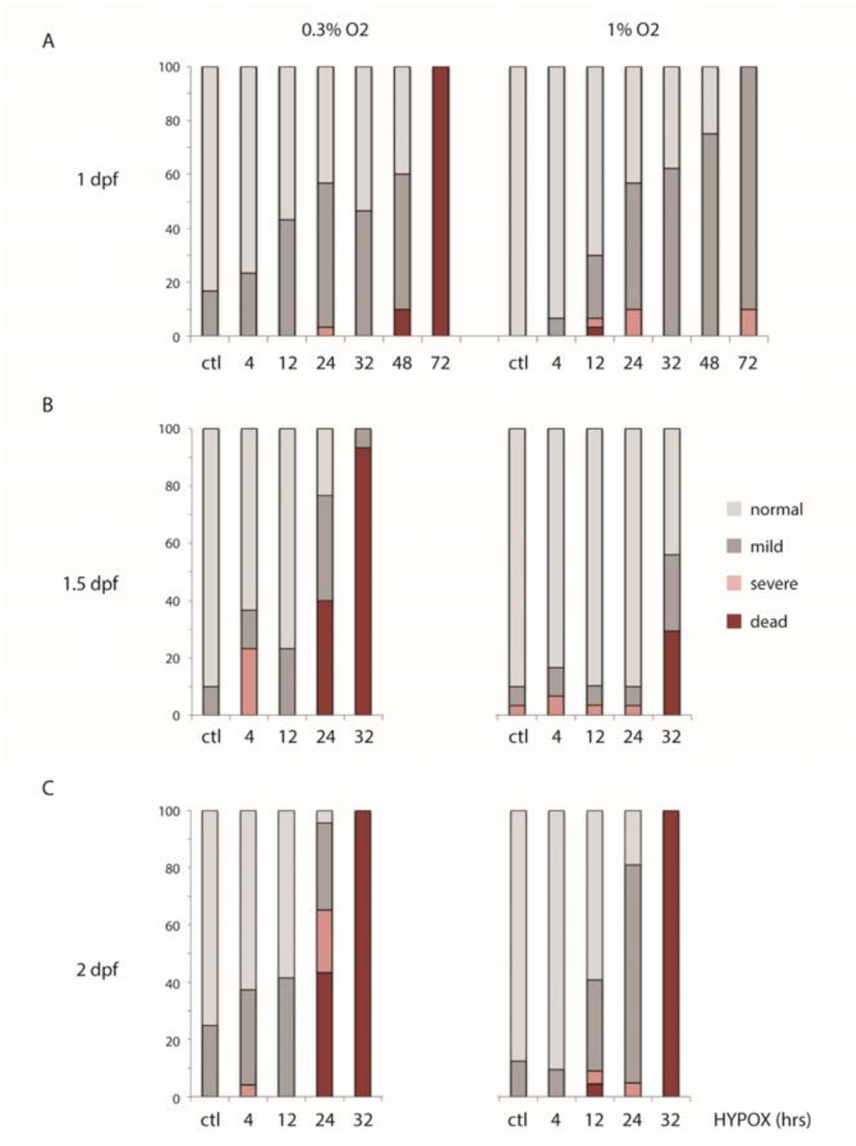

Figure S1. Hypoxia tolerance varies with developmental time and severity of stress. (A-C) Phenotypic categorization and survival of embryos subjected to increasing durations of hypoxic stress at either 0.3% or 1% oxygen. The milder 1% oxygen exposure is better tolerated, as is exposure earlier in development, demonstrated by increased abnormal and/or dead embryos for the same duration of hypoxia initiated in older embryos. Mean of n = 2 biological replicates presented per condition.

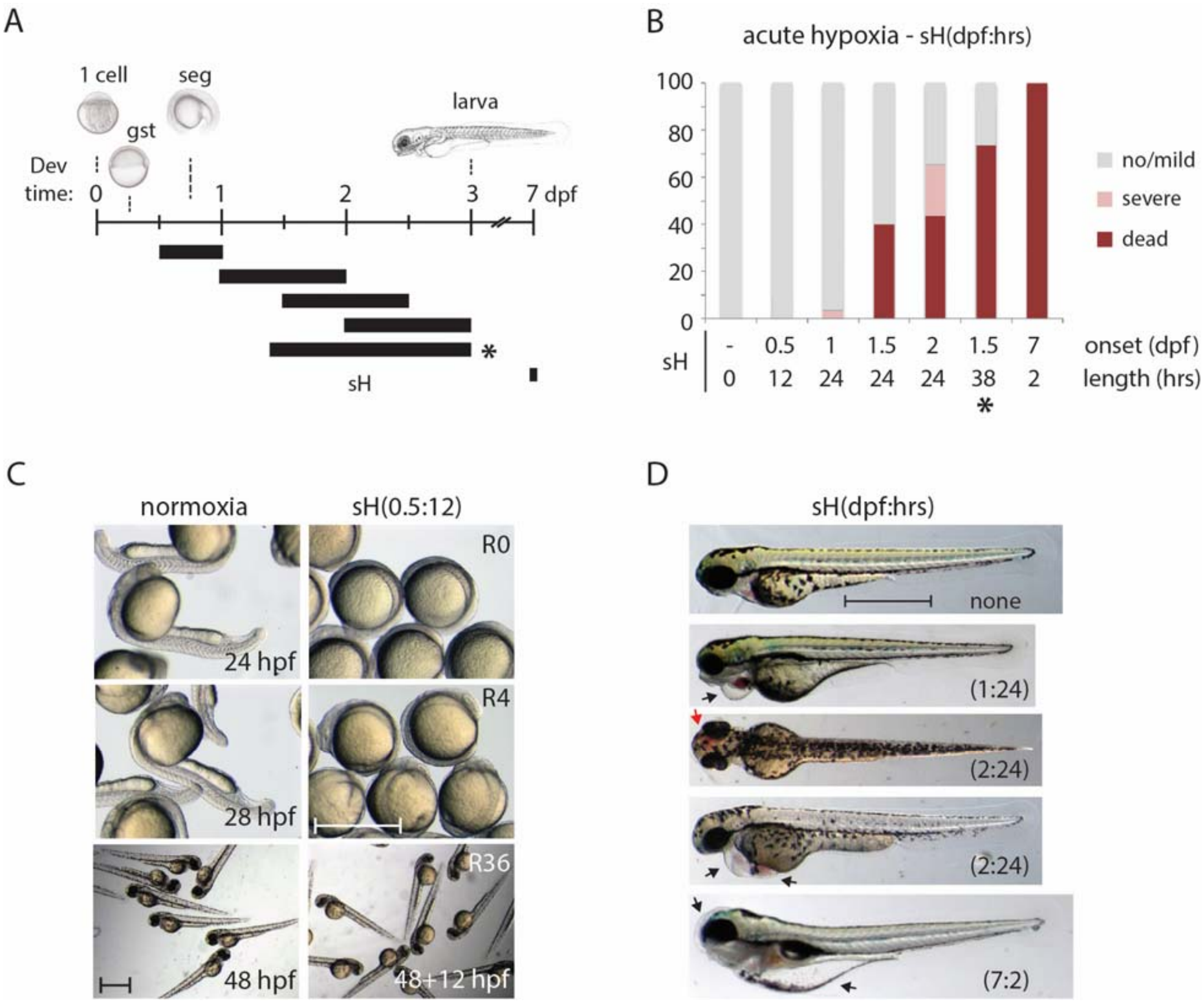

Figure S2. Embryonic and larval hypoxia exposure causes reversible developmental arrest and can cause diverse developmental defects. (A) Schematic indicating various developmental exposures to 0.3% oxygen at different times in development (sH; black bars). **(B)** Percentage distribution of phenotypes from each hPC protocol as shown in (A), grouped by severity of defects at 5 dpf. Hypoxia tolerance is highest in early embryos, as exemplified by 100% survival of a 24-hour hypoxia exposure initiated at 1 dpf, sH(1d:24h), contrasted with 0% survival of a much shorter, 2-hour hypoxia exposure at 7 dpf, sH(7d:2h). **(C)** Embryos exposed to sH(0.5d:12h), shown at 0, 4, and 36 hours of recovery in comparison to normoxic controls. Hypoxia-exposed animals at 36 hours of recovery are developmentally delayed approximately 12 hours, consistent with the duration of severe hypoxia exposure. **(D)** Representative surviving larvae from indicated sH protocols demonstrate pericardial, yolk sac, and/or ocular edema (black arrows), brain hemorrhage (red arrow), and neuromuscular impairment indicated by failure to hatch or to respond to touch (not shown). Scale bar = 1 mm. n = 2 to 4 biological replicates with s.e.m. per condition for (B).

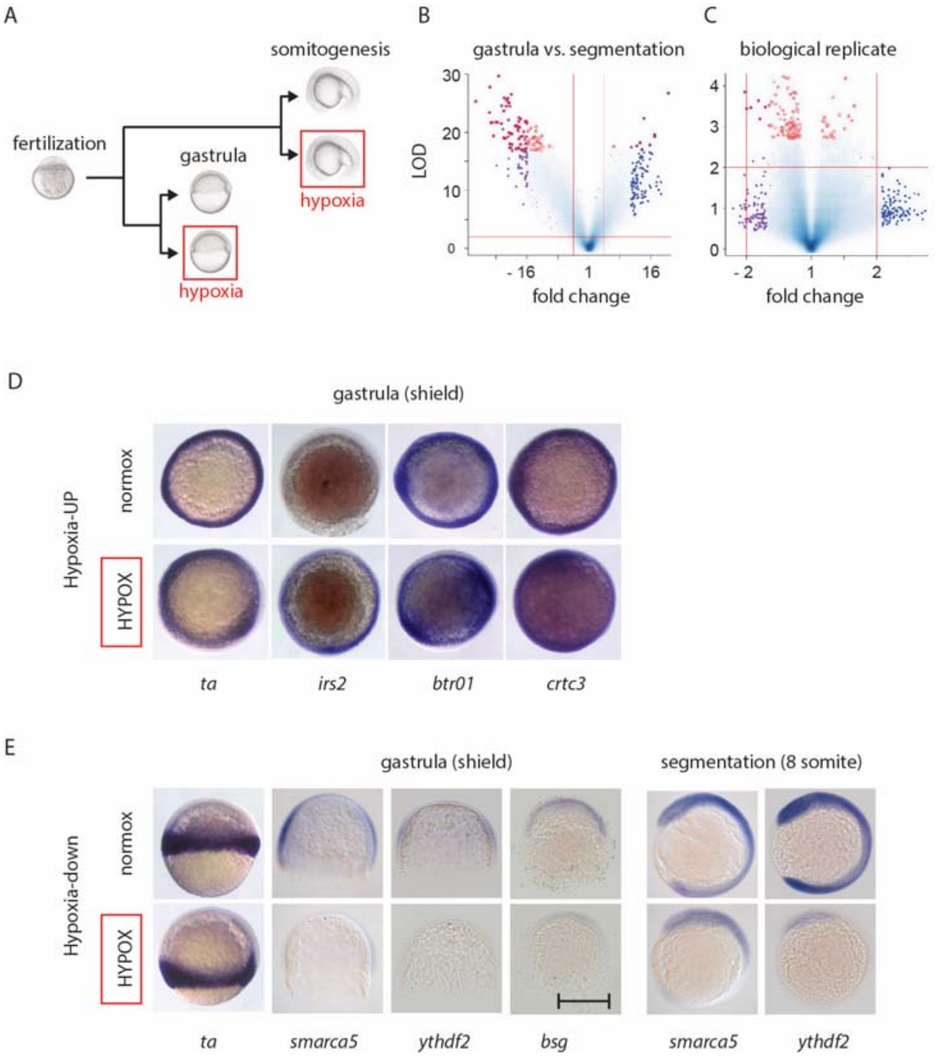

Figure S3. Validation of genome-wide hypoxia transcriptome screen. (A) Experimental approach for transcriptome analysis, with control and hypoxia-exposed samples obtained from gastrulation stage (shield) and segmentation stage embryos (8-somite). Differential gene expression was performed for control and hypoxia-exposed embryos at both stages using custom full-genome oligonucleotide microarrays. **(B, C)** Log-log volcano scatterplots of gene expression as in Fig. 2A, showing data for 26,259 genes. We performed further bioinformatic validation of genome-wide dataset via data permutation, where instead of grouping samples on the basis of hypoxic vs. normoxic exposure, we assigned them to two groups either by developmental stage (B, positive control) or by replicate group (C, negative control). As expected, comparison of gene expression between gastrula and segmentation stages, irrespective of stress, yielded hundreds of significantly differentially expressed genes, many of which related to cell fate determination and tissue differentiation. In contrast, comparison of experimental replicate groups 1 and 2 yielded the expected paucity of genes that passed significance and fold change criteria. **(D)** Hypoxia-upregulated microarray target gene validation shown via *in situ* hybridization at gastrula (shield) stage for *irs2, btr01*, and *crtc3* as compared to control gene *ta* (dorsal view). **(E)** Hypoxia-downregulated microarray target gene validation shown via *in situ* hybridization at gastrula (shield) and/or segmentation (8 somite) stages for *smarca5*, *ythdf2*, and *bsg* compared to control (lateral view). Scale bar = 0.5 mm.

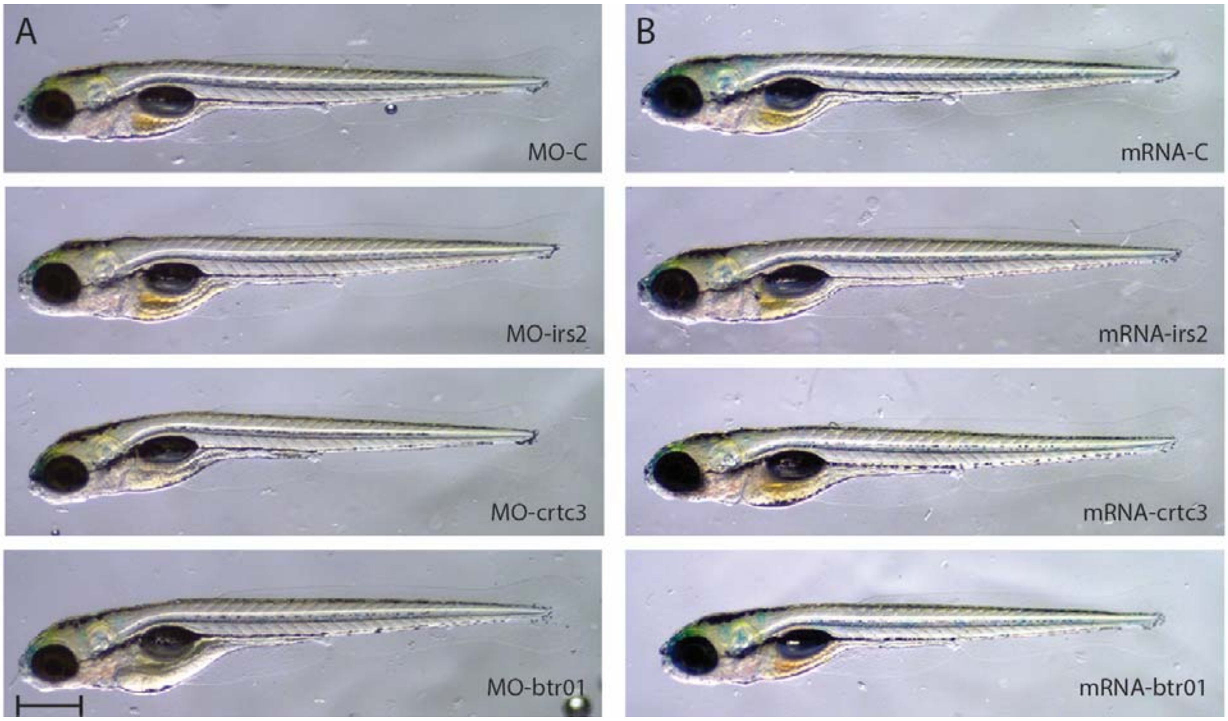

Figure S4. Identified hypoxia target genes *irs2*, *crtc3*, and *btr01* do not show knockdown or overexpression phenotypes under normoxia. (A) Representative ctl-MO and h-MO non-stressed morphant larvae shown at 5 dpf. **(B)** Representative ctl-mRNA and hypoxia target gene mRNA-injected, non-stressed larvae shown at 5 dpf. Scale bar = 0.5 mm.

Table S1 Top 100 hypoxia-induced transcripts.

	ID	logFC	AveExpr	P.Value	adj.P.Val	nuc_seq_for_search	zfin_searchterm
1	ENSDART00000099458	3.78	10.57	0.0009	0.0545	XM_001333606	ENSDARG00000068699
2	ENSDART00000104230	3.69	10.63	0.0025	0.0650	XR_029677	ENSDARG00000070779
3	ENSDART00000104902	3.68	10.06	0.0002	0.0419	ENSDART00000104902	ENSDARG00000056971
4	ENSDART00000059760	3.62	9.51	0.0000	0.0355	XM_695773	ENSDARG00000040780
5	ENSDART00000077091	3.53	10.98	0.0024	0.0641	XM_693874	ENSDARG00000024208
6	TC265422	3.52	9.70	0.0004	0.0455	TC358172	none
7	ENSDART00000063497	3.49	9.38	0.0004	0.0439	ENSDART00000063497	ENSDARG00000043255
8	ENSDART00000105335	3.45	10.78	0.0003	0.0428	XM_001338372	ENSDARG00000071299
9	OTTDART00000023331	3.45	10.80	0.0034	0.0701	XM_683394	OTTDARG00000019084
10	ENSDART00000079121	3.44	9.63	0.0000	0.0355	XM_694906	ENSDARG00000056617
11	ENSDART00000074956	3.42	9.51	0.0002	0.0417	ENSDART00000074956	ENSDARG00000053004
12	ENSDART00000099829	3.33	10.44	0.0003	0.0428	ENSDART00000099829	ENSDARG00000068911
13	ZV700S00006605	3.32	11.86	0.0021	0.0626	NM_213310	NM_213310
14	ENSDART00000099973	3.31	10.26	0.0013	0.0573	XM_687800	ENSDARG00000060875
15	ENSDART00000085244	3.28	9.66	0.0001	0.0380	ENSDART00000085244	ENSDARG00000068240
16	ENSDART00000079707	3.13	10.16	0.0005	0.0468	ENSDART00000079707	ENSDARG00000057103
17	ENSDART00000103629	3.13	9.13	0.0021	0.0623	XM_690067	ENSDARG00000070534
18	ENSDART00000097157	3.05	11.41	0.0019	0.0618	ENSDART00000097157	ENSDARG00000067515
19	ENSDART00000063770	3.05	9.88	0.0036	0.0715	XM_695043	ENSDARG00000004633
20	NM_199210	3.04	11.37	0.0034	0.0706	NM_199210	NM_199210
21	ENSDART00000103902	3.03	9.63	0.0009	0.0539	XM_691082	ENSDARG00000058478
22	ENSDART00000024625	3.03	10.54	0.0004	0.0442	XM_684551	ENSDARG00000003209
23	ENSDART00000060147	3.01	9.40	0.0009	0.0539	ENSDART00000060147	ENSDARG00000041050
24	TC249446	3.01	9.27	0.0001	0.0380	TC351285	BI980436
25	OTTDART00000007690	2.99	10.42	0.0002	0.0403	ENSDART00000054656	ENSDARG00000037534
26	ENSDART00000078652	2.98	9.91	0.0030	0.0675	XM_001332303	ENSDARG00000056206
27	ENSDART00000013266	2.90	9.32	0.0000	0.0355	ENSDART00000013266	ENSDARG00000011634
28	ZV700S00000733	2.82	9.12	0.0000	0.0355	NM_131830	NM_131830
29	ENSDART00000030593	2.82	9.68	0.0000	0.0355	XM_679135	ENSDARG00000002840
30	OTTDART00000011976	2.81	9.34	0.0001	0.0380	XM_701357	OTTDARG00000010407
31	ZV700S00003375	2.81	9.47	0.0018	0.0608	TC354929	BI885475
32	ENSDART00000104135	2.81	10.35	0.0003	0.0428	XM_001339475	ENSDARG00000070730
33	ENSDART00000103615	2.79	10.31	0.0002	0.0428	XM_001339894	ENSDARG00000070526
34	ENSDART00000104945	2.78	12.46	0.0018	0.0608	ENSDART00000104945	ENSDARG00000071125
35	AW232573	2.76	8.82	0.0007	0.0527	AW232573	AW232573
36	ENSDART00000097156	2.76	9.67	0.0014	0.0575	ENSDART00000097156	ENSDARG00000067515
37	OTTDART00000024857	2.75	9.68	0.0009	0.0544	XM_679063	OTTDARG00000020010
38	ENSDART00000073900	2.73	9.07	0.0002	0.0419	XM_693452	ENSDARG00000052114
39	ENSDART00000106259	2.71	9.30	0.0005	0.0458	ENSDART00000106259	ENSDARG00000071725
40	ENSDART00000038611	2.69	10.14	0.0014	0.0575	XR_029090	ENSDARG00000027618
41	ENSDART00000103763	2.68	10.82	0.0027	0.0660	ENSDART00000103763	ENSDARG00000070576
42	CK705703	2.65	10.54	0.0002	0.0419	TC364684	CK705703
43	OTTDART00000028786	2.65	9.39	0.0001	0.0380	NM_200989	NM_200989
44	TC263482	2.64	9.43	0.0012	0.0558	NM_001044796	NM_001044796
45	ENSDART00000092677	2.64	12.21	0.0030	0.0676	ENSDART00000092677	ENSDARG00000070503
46	BM889656	2.58	12.48	0.0001	0.0403	BM889656	BM889656
47	ENSDART00000027294	2.58	9.17	0.0001	0.0380	XM_001333296	ENSDARG00000012642
48	ENSDART00000100305	2.58	8.95	0.0001	0.0380	XM_684558	ENSDARG00000069113
49	ENSDART00000104377	2.57	11.37	0.0005	0.0468	XM_001337516	ENSDARG00000070855
50	ENSDART00000009796	2.54	10.33	0.0039	0.0728	XM_694854	ENSDARG00000024966
51	ZV700S00005379	2.54	12.84	0.0035	0.0714	NM_001082921	NM_001082921
52	ENSDART00000032386	2.53	8.64	0.0000	0.0380	XR_029801	ENSDARG00000051729
53	ENSDART00000076452	2.51	9.38	0.0035	0.0711	XR_029614	ENSDARG00000054320

54	ENSDART00000061110	2.50	8.49	0.0024	0.0641	ENSDART00000061110	ENSDARG00000041690
55	OTTDART00000006660	2.49	9.33	0.0001	0.0380	XM_686957	OTTDARG00000006075
56	ENSDART00000051788	2.47	12.30	0.0007	0.0507	XM_001338246	ENSDARG00000035710
57	NM_001080800	2.44	10.11	0.0028	0.0671	NM_001080800	NM_001080800
58	ENSDART00000062292	2.44	10.37	0.0015	0.0589	XM_001345201	ENSDARG00000023903
59	ENSDART00000099765	2.43	9.01	0.0027	0.0661	ENSDART00000099765	ENSDARG00000044119
60	ENSDART00000004908	2.43	10.71	0.0010	0.0545	ENSDART00000004908	ENSDARG00000003046
61	ENSDART00000087636	2.42	9.22	0.0009	0.0539	XR_029945	ENSDARG00000061455
62	OTTDART00000020489	2.41	8.72	0.0003	0.0428	OTTDART00000020489	OTTDARG00000017009
63	ENSDART00000074679	2.41	9.26	0.0023	0.0632	XM_001344779	ENSDARG00000052637
64	ENSDART00000097877	2.40	10.23	0.0008	0.0527	XM_001339482	ENSDARG00000067922
65	ENSDART00000063334	2.39	9.50	0.0029	0.0672	ENSDART00000063334	ENSDARG00000071624
66	ENSDART00000073801	2.38	9.32	0.0003	0.0428	ENSDART00000073801	ENSDARG00000052054
67	NM_001003472	2.38	12.49	0.0018	0.0608	NM_001003472	NM_001003472
68	ENSDART00000034589	2.35	10.14	0.0008	0.0527	ENSDART00000034589	ENSDARG00000054944
69	ENSDART00000099250	2.34	8.89	0.0001	0.0403	ENSDART00000099250	ENSDARG00000068585
70	ZV700S00004340	2.33	11.77	0.0000	0.0355	NM_212810	NM_212810
71	OTTDART00000025921	2.32	8.72	0.0001	0.0380	NM_131721	NM_131721
72	ENSDART00000082740	2.31	8.83	0.0001	0.0403	XM_001337128	ENSDARG00000059483
73	ENSDART00000067430	2.31	10.03	0.0009	0.0545	XM_001345218	ENSDARG00000045875
74	ENSDART00000101918	2.31	8.35	0.0000	0.0355	ENSDART00000101918	ENSDARG00000069823
75	ZV700S00002986	2.30	9.87	0.0016	0.0598	XM_001344589	BQ618148
76	TC242881	2.29	9.61	0.0014	0.0573	TC327783	BG306431
77	OTTDART00000013987	2.27	8.43	0.0000	0.0355	XM_001338390	OTTDARG00000011978
78	ENSDART00000079368	2.27	9.84	0.0025	0.0651	XM_001345157	ENSDARG00000038945
79	ZV700S00003817	2.27	9.45	0.0032	0.0692	AW279774	AW279774
80	ENSDART00000106532	2.26	9.81	0.0035	0.0708	ENSDART00000106532	ENSDARG00000071831
81	OTTDART00000014750	2.26	9.18	0.0007	0.0511	OTTDART00000014750	OTTDARG00000012512
82	OTTDART00000023474	2.24	12.08	0.0003	0.0430	NM_213151	NM_213151
83	TC262595	2.24	12.31	0.0008	0.0528	XM_689295	ENSDARG00000041535
84	ZV700S00005021	2.24	9.79	0.0003	0.0428	TC355044	AI878379
85	ENSDART00000079043	2.22	8.68	0.0004	0.0439	NM_001123053	NM_001123053
86	ENSDART00000100253	2.21	9.93	0.0009	0.0539	ENSDART00000100253	ENSDARG00000069089
87	NM_001020665	2.21	10.56	0.0010	0.0546	NM_001020665	NM_001020665
88	OTTDART00000026652	2.21	9.81	0.0003	0.0428	NM_201134	NM_201134
89	ENSDART00000006922	2.19	9.52	0.0008	0.0527	NM_001111243	NM_001111243
90	OTTDART00000011434	2.19	9.23	0.0014	0.0575	XM_678201	OTTDARG00000009962
91	ENSDART00000092701	2.19	9.24	0.0037	0.0722	XR_029828	ENSDARG00000063420
92	OTTDART00000028300	2.18	9.60	0.0006	0.0489	NM_001007453	NM_001007453
93	ENSDART00000065849	2.18	8.86	0.0001	0.0380	XM_001341054	ENSDARG00000032885
94	ENSDART00000058590	2.17	8.67	0.0015	0.0582	XM_681493	ENSDARG00000018096
95	ENSDART00000097521	2.17	8.40	0.0002	0.0419	ENSDART00000097521	ENSDARG00000067710
96	ENSDART00000100639	2.17	9.33	0.0001	0.0397	ENSDART00000100639	ENSDARG00000069254
97	ZV700S00006504	2.16	10.14	0.0030	0.0677	XM_688347	BI476240
98	ENSDART00000023109	2.16	9.93	0.0033	0.0696	XM_001338241	ENSDARG00000010083
99	ENSDART00000077154	2.16	12.43	0.0035	0.0714	NM_212793	NM_212793
100	ENSDART00000088004	2.15	9.17	0.0036	0.0715	XM_686765	ENSDARG00000061603

Table S2 Top 100 hypoxia-repressed transcripts.

	ID	logFC	AveExpr	P.Value	adj.P.Val	nuc_seq_for_search	zfin_searchterm
1	BC047846.1	-3.02	11.44	0.0003	0.0428	NM_200250	NM_200250
2	OTTDART00000011891	-2.91	12.57	0.0004	0.0455	NM_198142	NM_198142
3	ZV700S00002766	-2.84	10.34	0.0011	0.0553	TC313894	AL717746
4	ZV700S00002581	-2.82	11.50	0.0008	0.0539	TC315769	BQ078334
5	ENSDART00000079913	-2.76	10.64	0.0004	0.0455	XM_001336773	ENSDARG00000057292
6	TC253816	-2.67	10.60	0.0000	0.0355	TC314880	CN173392
7	AI384218	-2.58	11.26	0.0017	0.0599	AI384218	AI384218
8	TC252139	-2.52	10.63	0.0018	0.0610	TC350751	CK397153
9	OTTDART00000020020	-2.49	10.40	0.0002	0.0403	NM_001033108	NM_001033108
10	TC246674	-2.49	9.28	0.0001	0.0380	TC339735	EH450933
11	OTTDART00000023577	-2.48	13.04	0.0020	0.0621	NM_201336	NM_201336
12	ZV700S00005193	-2.48	11.17	0.0005	0.0460	ENSDART00000032307	ENSDARG00000010437
13	ZV700S00001225	-2.48	9.51	0.0000	0.0355	NM_001076605	NM_001076605
14	ZV700S00001586	-2.47	9.61	0.0000	0.0380	TC314880	BM035571
15	TC259486	-2.44	12.89	0.0001	0.0403	TC313481	CO809934
16	ZV700S00002031	-2.39	11.01	0.0001	0.0380	NM_205681	NM_205681
17	OTTDART00000030361	-2.39	9.54	0.0000	0.0355	NM_001005595	NM_001005595
18	BI878415	-2.39	11.03	0.0039	0.0731	BI878415	BI878415
19	ZV700S00002485	-2.38	10.39	0.0036	0.0715	ENSDART00000104101	ENSDARG00000040238
20	TC252941	-2.37	10.24	0.0018	0.0610	TC311218	CT706283
21	OTTDART00000028263	-2.35	9.99	0.0035	0.0714	NM_001013450	NM_001013450
22	TC245407	-2.34	12.68	0.0005	0.0455	TC337470	BI866616
23	TC262641	-2.32	9.62	0.0003	0.0428	TC344166	EH600992
24	ZV700S00001778	-2.31	9.81	0.0003	0.0428	NM_001114314	NM_001114314
25	OTTDART00000023001	-2.30	10.93	0.0005	0.0463	NM_001083818	NM_001083818
26	ZV700S00001230	-2.30	9.30	0.0008	0.0539	NM_001003566	NM_001003566
27	ZV700S00000938	-2.28	10.37	0.0010	0.0545	NM_001002476	NM_001002476
28	ENSDART00000048570	-2.24	10.41	0.0014	0.0575	NM_001110281	NM_001110281
29	OTTDART00000022688	-2.23	11.87	0.0015	0.0582	NM_199755	NM_199755
30	OTTDART00000026006	-2.22	11.36	0.0000	0.0355	NM_200015	NM_200015
31	ENSDART00000102840	-2.22	11.07	0.0016	0.0594	NM_212730	NM_212730
32	ZV700S00003761	-2.18	9.88	0.0000	0.0355	AW077326	AW077326
33	ZV700S00001583	-2.18	11.08	0.0000	0.0355	NM_001003420	NM_001003420
34	OTTDART00000013765	-2.16	10.72	0.0004	0.0444	XM_692602	OTTDARG00000011797
35	AW280155	-2.15	11.70	0.0011	0.0550	AW280155	AW280155
36	ZV700S00000323	-2.14	9.09	0.0007	0.0517	TC343808	BM081103
37	ZV700S00004972	-2.12	11.42	0.0008	0.0538	NM_001002128	NM_001002128
38	OTTDART00000006094	-2.11	9.63	0.0001	0.0380	NM_001030160	NM_001030160
39	TC241853	-2.09	10.59	0.0005	0.0475	TC332841	AI957734
40	OTTDART00000025902	-2.06	9.27	0.0003	0.0428	XM_690867	OTTDARG00000020647
41	TC238179	-2.06	10.53	0.0006	0.0503	NM_131702	NM_131702
42	TC262148	-2.05	9.35	0.0011	0.0550	NM_001080562	NM_001080562
43	OTTDART00000011889	-2.05	12.07	0.0001	0.0392	NM_198142	NM_198142
44	OTTDART00000028156	-2.05	10.69	0.0013	0.0573	NM_200153	NM_200153
45	ZV700S00000317	-2.04	12.06	0.0034	0.0700	NM_200250	NM_200250
46	NM_200627	-2.03	10.88	0.0001	0.0380	NM_200627	NM_200627
47	ZV700S00006401	-2.03	9.10	0.0003	0.0428	NM_001030206	NM_001030206
48	TC261282	-2.00	13.20	0.0009	0.0544	TC319434	CK677004
49	TC258835	-1.99	11.38	0.0008	0.0535	ENSDART00000102304	ENSDARG00000069980
50	ZV700S00004125	-1.98	9.66	0.0016	0.0594	XM_679831	OTTDARG00000025878
51	ZV700S00003155	-1.97	9.45	0.0001	0.0380	NM_001002590	NM_001002590
52	OTTDART00000016696	-1.97	11.04	0.0037	0.0722	NM_001114315	NM_001114315
53	BC054639.1	-1.96	10.99	0.0003	0.0428	NM_131024	NM_131024

54	ZV700S00003519	-1.95	9.96	0.0022	0.0632	NM_213405	NM_213405
55	ENSDART00000074083	-1.94	10.08	0.0002	0.0417	XM_690199	ENSDARG00000052263
56	TC252463	-1.93	11.34	0.0015	0.0589	XM_687126	EH600028
57	ZV700S00003541	-1.93	9.51	0.0022	0.0631	TC311462	CT624217
58	NM_001005595	-1.93	11.88	0.0001	0.0380	NM_001005595	NM_001005595
59	NM_001002099	-1.93	10.74	0.0006	0.0487	NM_001002099	NM_001002099
60	OTTDART00000017714	-1.93	11.19	0.0017	0.0606	NM_212980	NM_212980
61	ENSDART00000098845	-1.93	11.86	0.0014	0.0581	XM_689779	ENSDARG00000068373
62	TC266406	-1.92	10.28	0.0001	0.0403	NM_001098770	NM_001098770
63	TC255518	-1.91	9.80	0.0001	0.0380	OTTDART00000030528	OTTDARG00000023438
64	ZV700S00000560	-1.90	11.42	0.0001	0.0380	BG728979	BG728979
65	NM_001006029	-1.89	10.86	0.0023	0.0633	NM_001006029	NM_001006029
66	OTTDART00000029310	-1.89	9.55	0.0020	0.0621	NM_200451	NM_200451
67	ZV700S00002519	-1.88	14.12	0.0000	0.0355	NM_001114410	NM_001114410
68	ENSDART00000078041	-1.88	9.79	0.0037	0.0721	XM_001344738	ENSDARG00000055650
69	ZV700S00005092	-1.88	9.13	0.0021	0.0624	NM_001005581	NM_001005581
70	OTTDART00000028144	-1.87	9.14	0.0012	0.0558	NM_001014290	NM_001014290
71	ZV700S00006443	-1.87	11.18	0.0007	0.0526	NM_001013544	NM_001013544
72	OTTDART00000025524	-1.86	11.07	0.0002	0.0417	NM_001014370	NM_001014370
73	ZV700S00006657	-1.86	10.07	0.0005	0.0475	NM_200535	NM_200535
74	ZV700S00005206	-1.85	12.56	0.0011	0.0551	NM_001002355	NM_001002355
75	ZV700S00001690	-1.84	10.67	0.0003	0.0430	NM_200246	NM_200246
76	OTTDART00000022942	-1.84	10.91	0.0030	0.0675	XM_001332920	OTTDARG00000018816
77	OTTDART00000025638	-1.83	11.38	0.0022	0.0632	NM_200675	NM_200675
78	OTTDART00000014856	-1.83	10.63	0.0014	0.0575	NM_212685	NM_212685
79	OTTDART00000017073	-1.82	9.57	0.0019	0.0611	NM_001020627	NM_001020627
80	ZV700S00005633	-1.82	9.61	0.0022	0.0632	TC339775	EE307048
81	ZV700S00003585	-1.82	9.25	0.0000	0.0355	TC358822	AI794155
82	OTTDART00000025065	-1.82	12.54	0.0015	0.0586	NM_198806	NM_198806
83	OTTDART00000024106	-1.81	11.23	0.0003	0.0428	NM_200640	NM_200640
84	OTTDART00000020565	-1.81	12.00	0.0013	0.0573	NM_001003747	NM_001003747
85	OTTDART00000010251	-1.80	10.39	0.0009	0.0539	NM_001007777	NM_001007777
86	ENSDART00000063874	-1.79	9.16	0.0034	0.0698	NM_200735	NM_200735
87	ENSDART00000103730	-1.79	10.02	0.0033	0.0695	NM_001007442	NM_001007442
88	OTTDART00000012701	-1.79	11.24	0.0001	0.0403	NM_212892	NM_212892
89	OTTDART00000025982	-1.79	10.65	0.0008	0.0527	NM_199799	NM_199799
90	OTTDART00000008409	-1.78	8.98	0.0021	0.0625	XM_001341725	OTTDARG00000007457
91	OTTDART00000024412	-1.78	14.43	0.0037	0.0722	NM_001009889	NM_001009889
92	OTTDART00000001751	-1.78	11.61	0.0005	0.0468	NM_200095	NM_200095
93	ZV700S00000253	-1.77	10.39	0.0013	0.0573	NM_153657	NM_153657
94	ZV700S00006144	-1.77	10.64	0.0006	0.0503	NM_001037430	NM_001037430
95	ZV700S00003410	-1.77	9.53	0.0006	0.0502	NM_205714	NM_205714
96	ZV700S00005911	-1.77	10.16	0.0001	0.0380	NM_001077730	NM_001077730
97	OTTDART00000027634	-1.77	11.31	0.0001	0.0396	XM_001336922	OTTDARG00000021657
98	OTTDART00000016734	-1.76	9.43	0.0012	0.0558	OTTDART00000016734	OTTDARG00000014045
99	OTTDART00000029581	-1.76	10.23	0.0000	0.0355	NM_200190	NM_200190
100	OTTDART00000029791	-1.75	9.68	0.0032	0.0692	NM_001024388	NM_001024388

Table S3 Morpholinos used.

Gene	MO phenotype	MO type	**Ensembl transcript ID** (MO predicted result)	MO sequence (5' to 3')
ttll11	none/mild	Splice, 1st EXON-intron	Retention of 14.2 kb intron (ENSDART00000085684)	AGTGGGTCCTGAGCTCTTACCTCTT
irs2	hPC, acute hypoxia-sensitive	ATG	Translational inhibition (ENSDART00000053924)	CCCCTTTAAGAGGCGGACTTGCCAT
btr01	hPC	Splice, 1st EXON-intron	Retention of 1.2 kb intron (ENSDART00000073689)	AACCGTATAAATGTGCTTACCTTCC
camk2g2	hPC	Splice, 1st EXON-intron	Retention of 10.8 kb intron (ENSDART00000078652)	GAACAGGACACTGAGACTCACCTAT
crtc3	hPC	Splice, 1st EXON-intron	Retention of 15.7 kb intron (ENSDART00000073903)	TCCTAATTTGGCTGAGCTTACCCTT
ncam2	hPC	Splice, 1st EXON-intron	Retention of 319.1 kb intron (ENSDART00000100681)	TCACGATCTCGCATAAATACCTTGA
pacsin3	developmental	ATG	Translational inhibition (ENSDART00000098296)	TGCAGATCACCGTTGGAAGACATTT
mical2b	developmental	ATG	Translational inhibition (ENSDART00000139013)	TCCGTTCTTCCTCCGTCTCCCCCAT
inhbb	developmental	Splice, 1st intron-EXON	Skip 2nd of 2 exons (ENSDART00000059762)	GCCGTCTGTGGAGAAAACACACACA
opn5	developmental	Splice, 3rd EXON-intron	Retention of 6.4 kb intron (ENSDART00000033404)	TGAAAACTGAATACTGCACCTTCAC

Table S4 Oligonucleotide primers used[1].

I. Primers used for qPCR studies. For primers overlapping splice sites, both involved exons are listed.

Primer	Sequence (5' to 3')	Exon
*egln3*_qP-f	GCATTCGTGCGAGGTCAAAGGC	1
*egln3*_qP-r	GCAATCCCCCATGTTCCTTGGCA	2-3
*ttll11*_qP-f	TCCCAGCATGAGGATTGAACACG	3
*ttll11*_qP-r	CCGGCATTTGACGTGATGTGTTTC	4-5
*camk2b*_qP-f	AGAGGCCCGGATCTGCCGTT	1
*camk2b*_qP-r	TGCTGGTGGATGTGGCTGACG	4
*btr01*_qP-f	CGGCCATACTGTGACGGAGGC	1
*btr01*_qP-r	CTGAGACCGCAGTTCGGCGG	3
*ncam2*_qP-f	TCGGATTGCTCGTCGGTGGC	1
*ncam2*_qP-r	GCCTGGCACCGGTAGATGCC	3
*crtc3*_qP-f	GCAGCTCATGACAGACCTCACTGT	1
*crtc3*_qP-r	TGGAAATCAGTGCTGGCATTGCCT	2
*irs2*_qP-f	ACACAGCTCTGCCTCCGTAGA	1
*irs2*_qP-r	GGAGTAACCTCTGCTTCCTGCTCA	2
*sept8a*_qP-f	CCTCGGCGGTCATGTTGGCTT	1-2
*sept8a*_qP-r	GTTCTGGGCCGCAGGTAGACG	3
*eef1a1l1*_qP-f	AGAAGGAAGCCGCTGAGATGG	3
*eef1a1l1*_qP-r	TCCGTTCTTGGAGATACCAGCC	4

II. Primers used for amplification and cloning of full-length mRNAs.

Primer	Sequence (5' to 3')
*irs2*_FL-f: <u>ClaI</u> / **Start**	GGTGGT<u>ATCGAT</u>**ATG**GCAAGTCCGCCTCTTA
*irs2*_FL-r: <u>XhoI</u> / **Stop**	GCCACC<u>CTCGAG</u>**TCA**ATCTTGTACAGTGGTTGCAG
*crtc3*_FL-f: <u>Cla</u> / **Start**	GGTGGT<u>ATCGAT</u>**ATG**TCTGGATCCCCGGGC
*crtc3*_FL-r: <u>XbaI</u> / **Stop**	GCCACC<u>TCTAGA</u>**CTA**AAGTCTGTCACTGCGAAAGC
*btr01*_FL-f: <u>Cla</u> / **Start**	GGTGGT<u>ATCGAT</u>**ATG**TCATTTCCTGGTGAATTCCTGTC
*btr01*_FL-r: <u>XbaI</u> / **Stop**	GCCACC<u>TCTAGA</u>**CTA**TTCTAACCAGCTTGGGTCAGC

[1] Relevant restriction sites are underlined, and start/stop codons are in bold.

III. Primers used for amplification and cloning of in situ hybridization probes. Primers containing restriction sites were directionally cloned into pCS2p+. Primers without restriction sites were TA cloned into vector pCRII and directionality was validated by sequencing.

**Primer**	**Sequence (5' to 3')**
*bsg*_IS-f: BamHI	ATGCGGATCCGGTTTCAAGCCGAAGCTATG
*bsg*_IS-r: XbaI	ATGCTCTAGAGACCCAATAGTGGCCTTTGA
*ythdf2*_IS-f: BamHI	ATGCGGATCCCAAACAAACGCGCAGTCTAA
*ythdf2*_IS-r: XbaI	ATGCTCTAGACAGTGCTCCAGACTGTCCAA
*smarca5*_IS-f: BamHI	ATGCGGATCCGGAGAGCGTTTACGATGAGC
*smarca5*_IS-r: XbaI	ATGCTCTAGACGACAGCACAGCCGTAGTTA
*irs2*_IS-f:	CTTCAGTCAGCCCCACTAAC
*irs2*_IS-r:	CCTGCTTTACAACAACCGCC
*crtc3*_IS-f:	GGACGTTTCCCTCAGGCCTG
*crtc3*_IS-r:	GGTAGTGGGACAGACCCGCG
*btr01*_IS-f:	CGTGCATTTCCTCCTACTGGG
*btr01*_IS-r:	GCAAACATACTGATGGTGCCTG

Table S5 Full-length cDNA sequence information. Differences between our full-length cDNA sequence and the Ensembl transcript are listed below.

Gene<br>**Reference transcript**	**Location of Base Change**	**Consequence**
irs2<br>ENSDART00000053924	NA	NA
btr01<br>ENSDART00000073689	1129, insertion<br>(45 nucleotides)	retention of 45 nucleotides of predicted intronic sequence at the exon-intron 11 boundary, with a resulting in-frame addition of 15 amino acids:<br>GTTTGCACCCTTCCACAGAA<br>AGATCGCTGGGAGTGTATCA<br>ACAAA
crtc3<br>ENSDART00000073903	435-440, deletion<br>(6 nucleotides)	splicing-mediated exclusion of the first 6 bases of predicted exon 5, with a resulting in-frame deletion of two amino acids:<br>CCACAG
crtc3<br>ENSDART00000073903	G1098A	synonymous variant (proline)
crtc3<br>ENSDART00000073903	1347, insertion<br>(45 nucleotides)	addition of 45 nucleotides from a small exon within intron 4 of the Ensembl gene structure for which there is established EST evidence (AL913705), resulting in the addition of 15 amino acids:<br>GCAGGTGGACAACAACAACA<br>ACAACAGCAGCAACAGCAGC<br>ACCAC

Table S6 Genes with expression most highly correlated to *egln3* under all conditions, n = 6.

**Transcript**	**EST evidence**	**Gene info**
ENSDART00000028950	XM_684484	*gabrr3*, gamma-aminobutyric acid (GABA) receptor, rho 3
NM_001077375	NM_001077375	*ttll11*, tubulin tyrosine ligase-like family, member 11
OTTDART00000014783	XM_001332019	*pde4c*, phosphodiesterase 4C, cAMP-specific
ENSDART00000073688	XM_686415	*btr01*, bloodthirsty-related gene family, member 1
OTTDART00000008953	OTTDART00000008953	*msrb3*, methionine sulfoxide reductase B3
ENSDART00000064860	XM_682340	*rbms2*, RNA binding motif, single stranded interacting protein

File S1

R/Bioconductor code for bioinformatic analyses

Available for download at www.g3journal.org/lookup/suppl/doi:10.1534/g3.115.018010/-/DC1
