# Supplementary material for: Novel Genes Critical for Hypoxic Preconditioning in Zebrafish Are Regulators of Insulin and Glucose Metabolism
Source: G3 (Bethesda). 2015 Apr 3;5(6):1107–16. doi: 10.1534/g3.115.018010 (PMC4478541; doi:10.1534/g3.115.018010)
Supplement: Supporting Information [file supp_g3.115.018010_FigureS1.pdf]

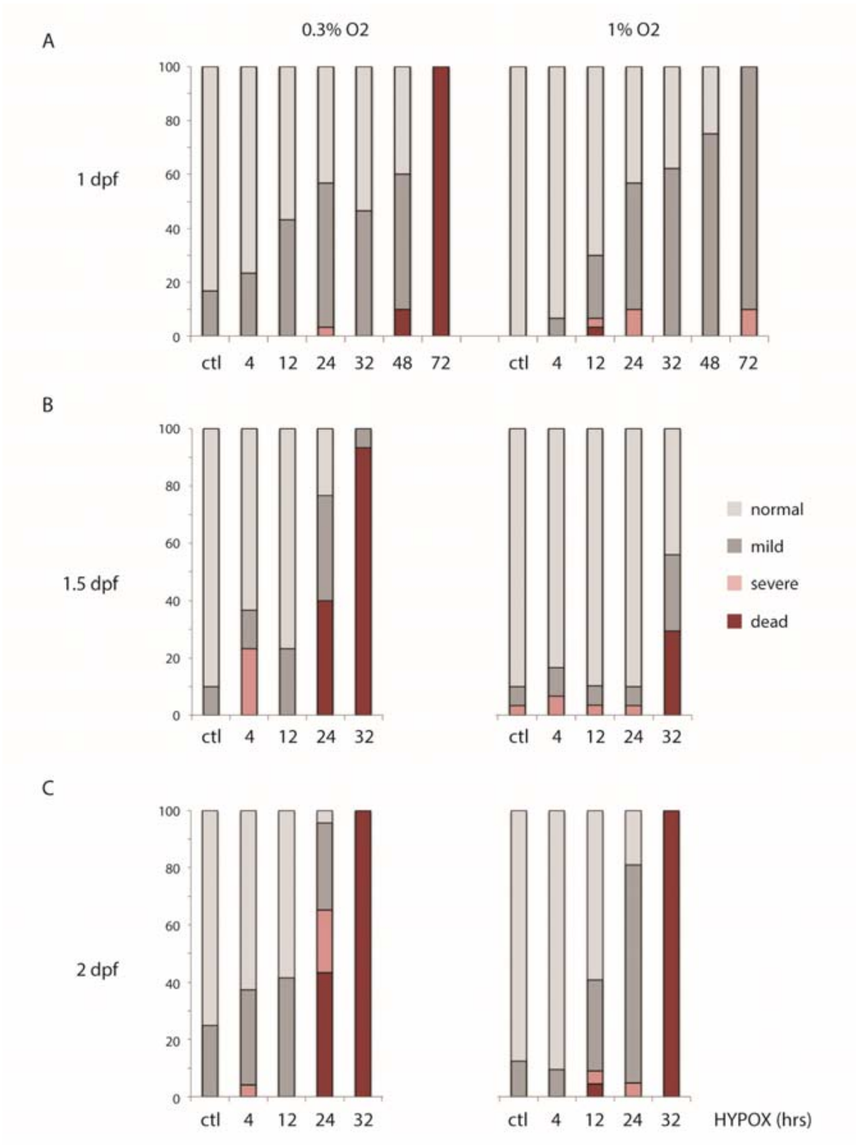

Figure S1. Hypoxia tolerance varies with developmental time and severity of stress. (A-C) Phenotypic categorization and survival of embryos subjected to increasing durations of hypoxic stress at either 0.3% or 1% oxygen. The milder 1% oxygen exposure is better tolerated, as is exposure earlier in development, demonstrated by increased abnormal and/or dead embryos for the same duration of hypoxia initiated in older embryos. Mean of n = 2 biological replicates presented per condition.
