# Supplementary material for: Novel Genes Critical for Hypoxic Preconditioning in Zebrafish Are Regulators of Insulin and Glucose Metabolism
Source: G3 (Bethesda). 2015 Apr 3;5(6):1107–16. doi: 10.1534/g3.115.018010 (PMC4478541; doi:10.1534/g3.115.018010)
Supplement: Supporting Information [file supp_g3.115.018010_FigureS2.pdf]

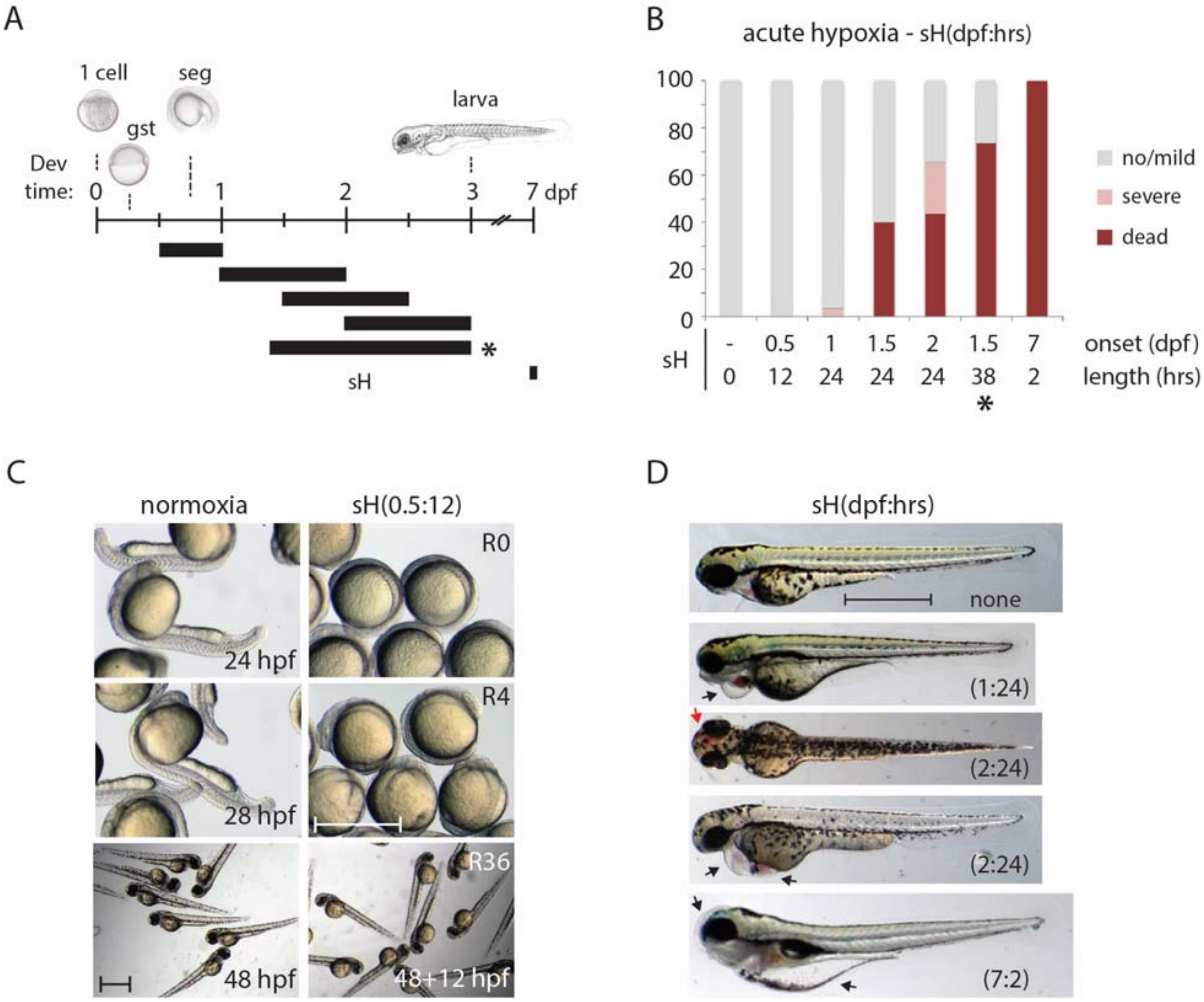

Figure S2. Embryonic and larval hypoxia exposure causes reversible developmental arrest and can cause diverse developmental defects. (A) Schematic indicating various developmental exposures to 0.3% oxygen at different times in development (sH; black bars). **(B)** Percentage distribution of phenotypes from each hPC protocol as shown in (A), grouped by severity of defects at 5 dpf. Hypoxia tolerance is highest in early embryos, as exemplified by 100% survival of a 24-hour hypoxia exposure initiated at 1 dpf, sH(1d:24h), contrasted with 0% survival of a much shorter, 2-hour hypoxia exposure at 7 dpf, sH(7d:2h). **(C)** Embryos exposed to sH(0.5d:12h), shown at 0, 4, and 36 hours of recovery in comparison to normoxic controls. Hypoxia-exposed animals at 36 hours of recovery are developmentally delayed approximately 12 hours, consistent with the duration of severe hypoxia exposure. **(D)** Representative surviving larvae from indicated sH protocols demonstrate pericardial, yolk sac, and/or ocular edema (black arrows), brain hemorrhage (red arrow), and neuromuscular impairment indicated by failure to hatch or to respond to touch (not shown). Scale bar = 1 mm. n = 2 to 4 biological replicates with s.e.m. per condition for (B).
