# Supplementary material for: Novel Genes Critical for Hypoxic Preconditioning in Zebrafish Are Regulators of Insulin and Glucose Metabolism
Source: G3 (Bethesda). 2015 Apr 3;5(6):1107–16. doi: 10.1534/g3.115.018010 (PMC4478541; doi:10.1534/g3.115.018010)
Supplement: Supporting Information [file supp_g3.115.018010_FigureS3.pdf]

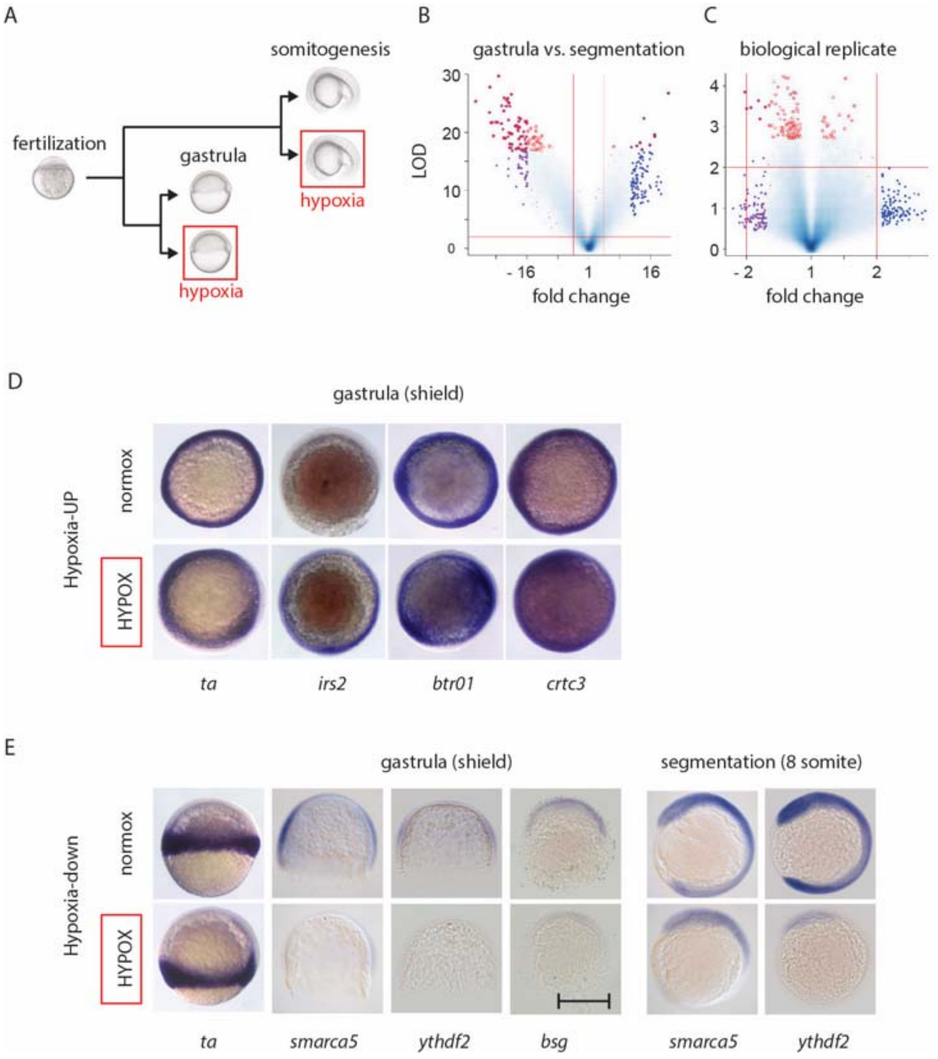

Figure S3. Validation of genome-wide hypoxia transcriptome screen. (A) Experimental approach for transcriptome analysis, with control and hypoxia-exposed samples obtained from gastrulation stage (shield) and segmentation stage embryos (8-somite). Differential gene expression was performed for control and hypoxia-exposed embryos at both stages using custom full-genome oligonucleotide microarrays. **(B, C)** Log-log volcano scatterplots of gene expression as in Fig. 2A, showing data for 26,259 genes. We performed further bioinformatic validation of genome-wide dataset via data permutation, where instead of grouping samples on the basis of hypoxic vs. normoxic exposure, we assigned them to two groups either by developmental stage (B, positive control) or by replicate group (C, negative control). As expected, comparison of gene expression between gastrula and segmentation stages, irrespective of stress, yielded hundreds of significantly differentially expressed genes, many of which related to cell fate determination and tissue differentiation. In contrast, comparison of experimental replicate groups 1 and 2 yielded the expected paucity of genes that passed significance and fold change criteria. **(D)** Hypoxia-upregulated microarray target gene validation shown via *in situ* hybridization at gastrula (shield) stage for *irs2, btr01*, and *crtc3* as compared to control gene *ta* (dorsal view). **(E)** Hypoxia-downregulated microarray target gene validation shown via *in situ* hybridization at gastrula (shield) and/or segmentation (8 somite) stages for *smarca5*, *ythdf2*, and *bsg* compared to control (lateral view). Scale bar = 0.5 mm.
