# Supplementary material for: Novel Genes Critical for Hypoxic Preconditioning in Zebrafish Are Regulators of Insulin and Glucose Metabolism
Source: G3 (Bethesda). 2015 Apr 3;5(6):1107–16. doi: 10.1534/g3.115.018010 (PMC4478541; doi:10.1534/g3.115.018010)
Supplement: Supporting Information [file supp_g3.115.018010_FigureS4.pdf]

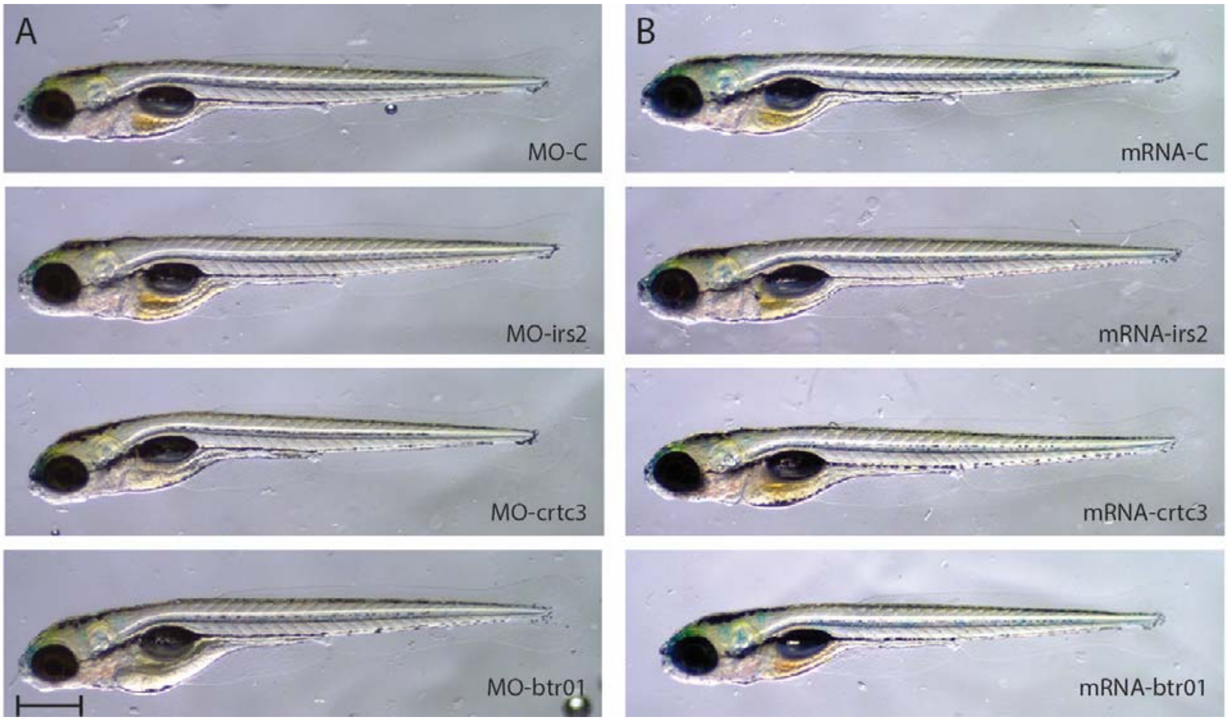

Figure S4. Identified hypoxia target genes *irs2*, *crtc3*, and *btr01* do not show knockdown or overexpression phenotypes under normoxia. (A) Representative ctl-MO and h-MO non-stressed morphant larvae shown at 5 dpf. **(B)** Representative ctl-mRNA and hypoxia target gene mRNA-injected, non-stressed larvae shown at 5 dpf. Scale bar = 0.5 mm.
