# Supplementary material for: Novel Genes Critical for Hypoxic Preconditioning in Zebrafish Are Regulators of Insulin and Glucose Metabolism
Source: G3 (Bethesda). 2015 Apr 3;5(6):1107–16. doi: 10.1534/g3.115.018010 (PMC4478541; doi:10.1534/g3.115.018010)
Supplement: Supporting Information [file supp_g3.115.018010_TableS1.pdf]

Table S1 Top 100 hypoxia-induced transcripts.

	ID	logFC	AveExpr	P.Value	adj.P.Val	nuc_seq_for_search	zfin_searchterm
1	ENSDART00000099458	3.78	10.57	0.0009	0.0545	XM_001333606	ENSDARG00000068699
2	ENSDART00000104230	3.69	10.63	0.0025	0.0650	XR_029677	ENSDARG00000070779
3	ENSDART00000104902	3.68	10.06	0.0002	0.0419	ENSDART00000104902	ENSDARG00000056971
4	ENSDART00000059760	3.62	9.51	0.0000	0.0355	XM_695773	ENSDARG00000040780
5	ENSDART00000077091	3.53	10.98	0.0024	0.0641	XM_693874	ENSDARG00000024208
6	TC265422	3.52	9.70	0.0004	0.0455	TC358172	none
7	ENSDART00000063497	3.49	9.38	0.0004	0.0439	ENSDART00000063497	ENSDARG00000043255
8	ENSDART00000105335	3.45	10.78	0.0003	0.0428	XM_001338372	ENSDARG00000071299
9	OTTDART00000023331	3.45	10.80	0.0034	0.0701	XM_683394	OTTDARG00000019084
10	ENSDART00000079121	3.44	9.63	0.0000	0.0355	XM_694906	ENSDARG00000056617
11	ENSDART00000074956	3.42	9.51	0.0002	0.0417	ENSDART00000074956	ENSDARG00000053004
12	ENSDART00000099829	3.33	10.44	0.0003	0.0428	ENSDART00000099829	ENSDARG00000068911
13	ZV700S00006605	3.32	11.86	0.0021	0.0626	NM_213310	NM_213310
14	ENSDART00000099973	3.31	10.26	0.0013	0.0573	XM_687800	ENSDARG00000060875
15	ENSDART00000085244	3.28	9.66	0.0001	0.0380	ENSDART00000085244	ENSDARG00000068240
16	ENSDART00000079707	3.13	10.16	0.0005	0.0468	ENSDART00000079707	ENSDARG00000057103
17	ENSDART00000103629	3.13	9.13	0.0021	0.0623	XM_690067	ENSDARG00000070534
18	ENSDART00000097157	3.05	11.41	0.0019	0.0618	ENSDART00000097157	ENSDARG00000067515
19	ENSDART00000063770	3.05	9.88	0.0036	0.0715	XM_695043	ENSDARG00000004633
20	NM_199210	3.04	11.37	0.0034	0.0706	NM_199210	NM_199210
21	ENSDART00000103902	3.03	9.63	0.0009	0.0539	XM_691082	ENSDARG00000058478
22	ENSDART00000024625	3.03	10.54	0.0004	0.0442	XM_684551	ENSDARG00000003209
23	ENSDART00000060147	3.01	9.40	0.0009	0.0539	ENSDART00000060147	ENSDARG00000041050
24	TC249446	3.01	9.27	0.0001	0.0380	TC351285	BI980436
25	OTTDART00000007690	2.99	10.42	0.0002	0.0403	ENSDART00000054656	ENSDARG00000037534
26	ENSDART00000078652	2.98	9.91	0.0030	0.0675	XM_001332303	ENSDARG00000056206
27	ENSDART00000013266	2.90	9.32	0.0000	0.0355	ENSDART00000013266	ENSDARG00000011634
28	ZV700S00000733	2.82	9.12	0.0000	0.0355	NM_131830	NM_131830
29	ENSDART00000030593	2.82	9.68	0.0000	0.0355	XM_679135	ENSDARG00000002840
30	OTTDART00000011976	2.81	9.34	0.0001	0.0380	XM_701357	OTTDARG00000010407
31	ZV700S00003375	2.81	9.47	0.0018	0.0608	TC354929	BI885475
32	ENSDART00000104135	2.81	10.35	0.0003	0.0428	XM_001339475	ENSDARG00000070730
33	ENSDART00000103615	2.79	10.31	0.0002	0.0428	XM_001339894	ENSDARG00000070526
34	ENSDART00000104945	2.78	12.46	0.0018	0.0608	ENSDART00000104945	ENSDARG00000071125
35	AW232573	2.76	8.82	0.0007	0.0527	AW232573	AW232573
36	ENSDART00000097156	2.76	9.67	0.0014	0.0575	ENSDART00000097156	ENSDARG00000067515
37	OTTDART00000024857	2.75	9.68	0.0009	0.0544	XM_679063	OTTDARG00000020010
38	ENSDART00000073900	2.73	9.07	0.0002	0.0419	XM_693452	ENSDARG00000052114
39	ENSDART00000106259	2.71	9.30	0.0005	0.0458	ENSDART00000106259	ENSDARG00000071725
40	ENSDART00000038611	2.69	10.14	0.0014	0.0575	XR_029090	ENSDARG00000027618
41	ENSDART00000103763	2.68	10.82	0.0027	0.0660	ENSDART00000103763	ENSDARG00000070576
42	CK705703	2.65	10.54	0.0002	0.0419	TC364684	CK705703
43	OTTDART00000028786	2.65	9.39	0.0001	0.0380	NM_200989	NM_200989
44	TC263482	2.64	9.43	0.0012	0.0558	NM_001044796	NM_001044796
45	ENSDART00000092677	2.64	12.21	0.0030	0.0676	ENSDART00000092677	ENSDARG00000070503
46	BM889656	2.58	12.48	0.0001	0.0403	BM889656	BM889656
47	ENSDART00000027294	2.58	9.17	0.0001	0.0380	XM_001333296	ENSDARG00000012642
48	ENSDART00000100305	2.58	8.95	0.0001	0.0380	XM_684558	ENSDARG00000069113
49	ENSDART00000104377	2.57	11.37	0.0005	0.0468	XM_001337516	ENSDARG00000070855
50	ENSDART00000009796	2.54	10.33	0.0039	0.0728	XM_694854	ENSDARG00000024966
51	ZV700S00005379	2.54	12.84	0.0035	0.0714	NM_001082921	NM_001082921
52	ENSDART00000032386	2.53	8.64	0.0000	0.0380	XR_029801	ENSDARG00000051729
53	ENSDART00000076452	2.51	9.38	0.0035	0.0711	XR_029614	ENSDARG00000054320

54	ENSDART00000061110	2.50	8.49	0.0024	0.0641	ENSDART00000061110	ENSDARG00000041690
55	OTTDART00000006660	2.49	9.33	0.0001	0.0380	XM_686957	OTTDARG00000006075
56	ENSDART00000051788	2.47	12.30	0.0007	0.0507	XM_001338246	ENSDARG00000035710
57	NM_001080800	2.44	10.11	0.0028	0.0671	NM_001080800	NM_001080800
58	ENSDART00000062292	2.44	10.37	0.0015	0.0589	XM_001345201	ENSDARG00000023903
59	ENSDART00000099765	2.43	9.01	0.0027	0.0661	ENSDART00000099765	ENSDARG00000044119
60	ENSDART00000004908	2.43	10.71	0.0010	0.0545	ENSDART00000004908	ENSDARG00000003046
61	ENSDART00000087636	2.42	9.22	0.0009	0.0539	XR_029945	ENSDARG00000061455
62	OTTDART00000020489	2.41	8.72	0.0003	0.0428	OTTDART00000020489	OTTDARG00000017009
63	ENSDART00000074679	2.41	9.26	0.0023	0.0632	XM_001344779	ENSDARG00000052637
64	ENSDART00000097877	2.40	10.23	0.0008	0.0527	XM_001339482	ENSDARG00000067922
65	ENSDART00000063334	2.39	9.50	0.0029	0.0672	ENSDART00000063334	ENSDARG00000071624
66	ENSDART00000073801	2.38	9.32	0.0003	0.0428	ENSDART00000073801	ENSDARG00000052054
67	NM_001003472	2.38	12.49	0.0018	0.0608	NM_001003472	NM_001003472
68	ENSDART00000034589	2.35	10.14	0.0008	0.0527	ENSDART00000034589	ENSDARG00000054944
69	ENSDART00000099250	2.34	8.89	0.0001	0.0403	ENSDART00000099250	ENSDARG00000068585
70	ZV700S00004340	2.33	11.77	0.0000	0.0355	NM_212810	NM_212810
71	OTTDART00000025921	2.32	8.72	0.0001	0.0380	NM_131721	NM_131721
72	ENSDART00000082740	2.31	8.83	0.0001	0.0403	XM_001337128	ENSDARG00000059483
73	ENSDART00000067430	2.31	10.03	0.0009	0.0545	XM_001345218	ENSDARG00000045875
74	ENSDART00000101918	2.31	8.35	0.0000	0.0355	ENSDART00000101918	ENSDARG00000069823
75	ZV700S00002986	2.30	9.87	0.0016	0.0598	XM_001344589	BQ618148
76	TC242881	2.29	9.61	0.0014	0.0573	TC327783	BG306431
77	OTTDART00000013987	2.27	8.43	0.0000	0.0355	XM_001338390	OTTDARG00000011978
78	ENSDART00000079368	2.27	9.84	0.0025	0.0651	XM_001345157	ENSDARG00000038945
79	ZV700S00003817	2.27	9.45	0.0032	0.0692	AW279774	AW279774
80	ENSDART00000106532	2.26	9.81	0.0035	0.0708	ENSDART00000106532	ENSDARG00000071831
81	OTTDART00000014750	2.26	9.18	0.0007	0.0511	OTTDART00000014750	OTTDARG00000012512
82	OTTDART00000023474	2.24	12.08	0.0003	0.0430	NM_213151	NM_213151
83	TC262595	2.24	12.31	0.0008	0.0528	XM_689295	ENSDARG00000041535
84	ZV700S00005021	2.24	9.79	0.0003	0.0428	TC355044	AI878379
85	ENSDART00000079043	2.22	8.68	0.0004	0.0439	NM_001123053	NM_001123053
86	ENSDART00000100253	2.21	9.93	0.0009	0.0539	ENSDART00000100253	ENSDARG00000069089
87	NM_001020665	2.21	10.56	0.0010	0.0546	NM_001020665	NM_001020665
88	OTTDART00000026652	2.21	9.81	0.0003	0.0428	NM_201134	NM_201134
89	ENSDART00000006922	2.19	9.52	0.0008	0.0527	NM_001111243	NM_001111243
90	OTTDART00000011434	2.19	9.23	0.0014	0.0575	XM_678201	OTTDARG00000009962
91	ENSDART00000092701	2.19	9.24	0.0037	0.0722	XR_029828	ENSDARG00000063420
92	OTTDART00000028300	2.18	9.60	0.0006	0.0489	NM_001007453	NM_001007453
93	ENSDART00000065849	2.18	8.86	0.0001	0.0380	XM_001341054	ENSDARG00000032885
94	ENSDART00000058590	2.17	8.67	0.0015	0.0582	XM_681493	ENSDARG00000018096
95	ENSDART00000097521	2.17	8.40	0.0002	0.0419	ENSDART00000097521	ENSDARG00000067710
96	ENSDART00000100639	2.17	9.33	0.0001	0.0397	ENSDART00000100639	ENSDARG00000069254
97	ZV700S00006504	2.16	10.14	0.0030	0.0677	XM_688347	BI476240
98	ENSDART00000023109	2.16	9.93	0.0033	0.0696	XM_001338241	ENSDARG00000010083
99	ENSDART00000077154	2.16	12.43	0.0035	0.0714	NM_212793	NM_212793
100	ENSDART00000088004	2.15	9.17	0.0036	0.0715	XM_686765	ENSDARG00000061603
