# Supplementary material for: Novel Genes Critical for Hypoxic Preconditioning in Zebrafish Are Regulators of Insulin and Glucose Metabolism
Source: G3 (Bethesda). 2015 Apr 3;5(6):1107–16. doi: 10.1534/g3.115.018010 (PMC4478541; doi:10.1534/g3.115.018010)
Supplement: Supporting Information [file supp_g3.115.018010_TableS2.pdf]

Table S2 Top 100 hypoxia-repressed transcripts.

	ID	logFC	AveExpr	P.Value	adj.P.Val	nuc_seq_for_search	zfin_searchterm
1	BC047846.1	-3.02	11.44	0.0003	0.0428	NM_200250	NM_200250
2	OTTDART00000011891	-2.91	12.57	0.0004	0.0455	NM_198142	NM_198142
3	ZV700S00002766	-2.84	10.34	0.0011	0.0553	TC313894	AL717746
4	ZV700S00002581	-2.82	11.50	0.0008	0.0539	TC315769	BQ078334
5	ENSDART00000079913	-2.76	10.64	0.0004	0.0455	XM_001336773	ENSDARG00000057292
6	TC253816	-2.67	10.60	0.0000	0.0355	TC314880	CN173392
7	AI384218	-2.58	11.26	0.0017	0.0599	AI384218	AI384218
8	TC252139	-2.52	10.63	0.0018	0.0610	TC350751	CK397153
9	OTTDART00000020020	-2.49	10.40	0.0002	0.0403	NM_001033108	NM_001033108
10	TC246674	-2.49	9.28	0.0001	0.0380	TC339735	EH450933
11	OTTDART00000023577	-2.48	13.04	0.0020	0.0621	NM_201336	NM_201336
12	ZV700S00005193	-2.48	11.17	0.0005	0.0460	ENSDART00000032307	ENSDARG00000010437
13	ZV700S00001225	-2.48	9.51	0.0000	0.0355	NM_001076605	NM_001076605
14	ZV700S00001586	-2.47	9.61	0.0000	0.0380	TC314880	BM035571
15	TC259486	-2.44	12.89	0.0001	0.0403	TC313481	CO809934
16	ZV700S00002031	-2.39	11.01	0.0001	0.0380	NM_205681	NM_205681
17	OTTDART00000030361	-2.39	9.54	0.0000	0.0355	NM_001005595	NM_001005595
18	BI878415	-2.39	11.03	0.0039	0.0731	BI878415	BI878415
19	ZV700S00002485	-2.38	10.39	0.0036	0.0715	ENSDART00000104101	ENSDARG00000040238
20	TC252941	-2.37	10.24	0.0018	0.0610	TC311218	CT706283
21	OTTDART00000028263	-2.35	9.99	0.0035	0.0714	NM_001013450	NM_001013450
22	TC245407	-2.34	12.68	0.0005	0.0455	TC337470	BI866616
23	TC262641	-2.32	9.62	0.0003	0.0428	TC344166	EH600992
24	ZV700S00001778	-2.31	9.81	0.0003	0.0428	NM_001114314	NM_001114314
25	OTTDART00000023001	-2.30	10.93	0.0005	0.0463	NM_001083818	NM_001083818
26	ZV700S00001230	-2.30	9.30	0.0008	0.0539	NM_001003566	NM_001003566
27	ZV700S00000938	-2.28	10.37	0.0010	0.0545	NM_001002476	NM_001002476
28	ENSDART00000048570	-2.24	10.41	0.0014	0.0575	NM_001110281	NM_001110281
29	OTTDART00000022688	-2.23	11.87	0.0015	0.0582	NM_199755	NM_199755
30	OTTDART00000026006	-2.22	11.36	0.0000	0.0355	NM_200015	NM_200015
31	ENSDART00000102840	-2.22	11.07	0.0016	0.0594	NM_212730	NM_212730
32	ZV700S00003761	-2.18	9.88	0.0000	0.0355	AW077326	AW077326
33	ZV700S00001583	-2.18	11.08	0.0000	0.0355	NM_001003420	NM_001003420
34	OTTDART00000013765	-2.16	10.72	0.0004	0.0444	XM_692602	OTTDARG00000011797
35	AW280155	-2.15	11.70	0.0011	0.0550	AW280155	AW280155
36	ZV700S00000323	-2.14	9.09	0.0007	0.0517	TC343808	BM081103
37	ZV700S00004972	-2.12	11.42	0.0008	0.0538	NM_001002128	NM_001002128
38	OTTDART00000006094	-2.11	9.63	0.0001	0.0380	NM_001030160	NM_001030160
39	TC241853	-2.09	10.59	0.0005	0.0475	TC332841	AI957734
40	OTTDART00000025902	-2.06	9.27	0.0003	0.0428	XM_690867	OTTDARG00000020647
41	TC238179	-2.06	10.53	0.0006	0.0503	NM_131702	NM_131702
42	TC262148	-2.05	9.35	0.0011	0.0550	NM_001080562	NM_001080562
43	OTTDART00000011889	-2.05	12.07	0.0001	0.0392	NM_198142	NM_198142
44	OTTDART00000028156	-2.05	10.69	0.0013	0.0573	NM_200153	NM_200153
45	ZV700S00000317	-2.04	12.06	0.0034	0.0700	NM_200250	NM_200250
46	NM_200627	-2.03	10.88	0.0001	0.0380	NM_200627	NM_200627
47	ZV700S00006401	-2.03	9.10	0.0003	0.0428	NM_001030206	NM_001030206
48	TC261282	-2.00	13.20	0.0009	0.0544	TC319434	CK677004
49	TC258835	-1.99	11.38	0.0008	0.0535	ENSDART00000102304	ENSDARG00000069980
50	ZV700S00004125	-1.98	9.66	0.0016	0.0594	XM_679831	OTTDARG00000025878
51	ZV700S00003155	-1.97	9.45	0.0001	0.0380	NM_001002590	NM_001002590
52	OTTDART00000016696	-1.97	11.04	0.0037	0.0722	NM_001114315	NM_001114315
53	BC054639.1	-1.96	10.99	0.0003	0.0428	NM_131024	NM_131024

54	ZV700S00003519	-1.95	9.96	0.0022	0.0632	NM_213405	NM_213405
55	ENSDART00000074083	-1.94	10.08	0.0002	0.0417	XM_690199	ENSDARG00000052263
56	TC252463	-1.93	11.34	0.0015	0.0589	XM_687126	EH600028
57	ZV700S00003541	-1.93	9.51	0.0022	0.0631	TC311462	CT624217
58	NM_001005595	-1.93	11.88	0.0001	0.0380	NM_001005595	NM_001005595
59	NM_001002099	-1.93	10.74	0.0006	0.0487	NM_001002099	NM_001002099
60	OTTDART00000017714	-1.93	11.19	0.0017	0.0606	NM_212980	NM_212980
61	ENSDART00000098845	-1.93	11.86	0.0014	0.0581	XM_689779	ENSDARG00000068373
62	TC266406	-1.92	10.28	0.0001	0.0403	NM_001098770	NM_001098770
63	TC255518	-1.91	9.80	0.0001	0.0380	OTTDART00000030528	OTTDARG00000023438
64	ZV700S00000560	-1.90	11.42	0.0001	0.0380	BG728979	BG728979
65	NM_001006029	-1.89	10.86	0.0023	0.0633	NM_001006029	NM_001006029
66	OTTDART00000029310	-1.89	9.55	0.0020	0.0621	NM_200451	NM_200451
67	ZV700S00002519	-1.88	14.12	0.0000	0.0355	NM_001114410	NM_001114410
68	ENSDART00000078041	-1.88	9.79	0.0037	0.0721	XM_001344738	ENSDARG00000055650
69	ZV700S00005092	-1.88	9.13	0.0021	0.0624	NM_001005581	NM_001005581
70	OTTDART00000028144	-1.87	9.14	0.0012	0.0558	NM_001014290	NM_001014290
71	ZV700S00006443	-1.87	11.18	0.0007	0.0526	NM_001013544	NM_001013544
72	OTTDART00000025524	-1.86	11.07	0.0002	0.0417	NM_001014370	NM_001014370
73	ZV700S00006657	-1.86	10.07	0.0005	0.0475	NM_200535	NM_200535
74	ZV700S00005206	-1.85	12.56	0.0011	0.0551	NM_001002355	NM_001002355
75	ZV700S00001690	-1.84	10.67	0.0003	0.0430	NM_200246	NM_200246
76	OTTDART00000022942	-1.84	10.91	0.0030	0.0675	XM_001332920	OTTDARG00000018816
77	OTTDART00000025638	-1.83	11.38	0.0022	0.0632	NM_200675	NM_200675
78	OTTDART00000014856	-1.83	10.63	0.0014	0.0575	NM_212685	NM_212685
79	OTTDART00000017073	-1.82	9.57	0.0019	0.0611	NM_001020627	NM_001020627
80	ZV700S00005633	-1.82	9.61	0.0022	0.0632	TC339775	EE307048
81	ZV700S00003585	-1.82	9.25	0.0000	0.0355	TC358822	AI794155
82	OTTDART00000025065	-1.82	12.54	0.0015	0.0586	NM_198806	NM_198806
83	OTTDART00000024106	-1.81	11.23	0.0003	0.0428	NM_200640	NM_200640
84	OTTDART00000020565	-1.81	12.00	0.0013	0.0573	NM_001003747	NM_001003747
85	OTTDART00000010251	-1.80	10.39	0.0009	0.0539	NM_001007777	NM_001007777
86	ENSDART00000063874	-1.79	9.16	0.0034	0.0698	NM_200735	NM_200735
87	ENSDART00000103730	-1.79	10.02	0.0033	0.0695	NM_001007442	NM_001007442
88	OTTDART00000012701	-1.79	11.24	0.0001	0.0403	NM_212892	NM_212892
89	OTTDART00000025982	-1.79	10.65	0.0008	0.0527	NM_199799	NM_199799
90	OTTDART00000008409	-1.78	8.98	0.0021	0.0625	XM_001341725	OTTDARG00000007457
91	OTTDART00000024412	-1.78	14.43	0.0037	0.0722	NM_001009889	NM_001009889
92	OTTDART00000001751	-1.78	11.61	0.0005	0.0468	NM_200095	NM_200095
93	ZV700S00000253	-1.77	10.39	0.0013	0.0573	NM_153657	NM_153657
94	ZV700S00006144	-1.77	10.64	0.0006	0.0503	NM_001037430	NM_001037430
95	ZV700S00003410	-1.77	9.53	0.0006	0.0502	NM_205714	NM_205714
96	ZV700S00005911	-1.77	10.16	0.0001	0.0380	NM_001077730	NM_001077730
97	OTTDART00000027634	-1.77	11.31	0.0001	0.0396	XM_001336922	OTTDARG00000021657
98	OTTDART00000016734	-1.76	9.43	0.0012	0.0558	OTTDART00000016734	OTTDARG00000014045
99	OTTDART00000029581	-1.76	10.23	0.0000	0.0355	NM_200190	NM_200190
100	OTTDART00000029791	-1.75	9.68	0.0032	0.0692	NM_001024388	NM_001024388
