# Supplementary material for: Novel Genes Critical for Hypoxic Preconditioning in Zebrafish Are Regulators of Insulin and Glucose Metabolism
Source: G3 (Bethesda). 2015 Apr 3;5(6):1107–16. doi: 10.1534/g3.115.018010 (PMC4478541; doi:10.1534/g3.115.018010)
Supplement: Supporting Information [file supp_g3.115.018010_TableS3.pdf]

Table S3 Morpholinos used.

Gene	MO phenotype	MO type	**Ensembl transcript ID** (MO predicted result)	MO sequence (5' to 3')
ttll11	none/mild	Splice, 1st EXON-intron	Retention of 14.2 kb intron (ENSDART00000085684)	AGTGGGTCCTGAGCTCTTACCTCTT
irs2	hPC, acute hypoxia-sensitive	ATG	Translational inhibition (ENSDART00000053924)	CCCCTTTAAGAGGCGGACTTGCCAT
btr01	hPC	Splice, 1st EXON-intron	Retention of 1.2 kb intron (ENSDART00000073689)	AACCGTATAAATGTGCTTACCTTCC
camk2g2	hPC	Splice, 1st EXON-intron	Retention of 10.8 kb intron (ENSDART00000078652)	GAACAGGACACTGAGACTCACCTAT
crtc3	hPC	Splice, 1st EXON-intron	Retention of 15.7 kb intron (ENSDART00000073903)	TCCTAATTTGGCTGAGCTTACCCTT
ncam2	hPC	Splice, 1st EXON-intron	Retention of 319.1 kb intron (ENSDART00000100681)	TCACGATCTCGCATAAATACCTTGA
pacsin3	developmental	ATG	Translational inhibition (ENSDART00000098296)	TGCAGATCACCGTTGGAAGACATTT
mical2b	developmental	ATG	Translational inhibition (ENSDART00000139013)	TCCGTTCTTCCTCCGTCTCCCCCAT
inhbb	developmental	Splice, 1st intron-EXON	Skip 2nd of 2 exons (ENSDART00000059762)	GCCGTCTGTGGAGAAAACACACACA
opn5	developmental	Splice, 3rd EXON-intron	Retention of 6.4 kb intron (ENSDART00000033404)	TGAAAACTGAATACTGCACCTTCAC
