# Supplementary material for: Novel Genes Critical for Hypoxic Preconditioning in Zebrafish Are Regulators of Insulin and Glucose Metabolism
Source: G3 (Bethesda). 2015 Apr 3;5(6):1107–16. doi: 10.1534/g3.115.018010 (PMC4478541; doi:10.1534/g3.115.018010)
Supplement: Supporting Information [file supp_g3.115.018010_TableS4.pdf]

Table S4 Oligonucleotide primers used[1].

I. Primers used for qPCR studies. For primers overlapping splice sites, both involved exons are listed.

**Primer**	**Sequence (5' to 3')**	**Exon**
*egln3*_qP-f	GCATTCGTGCGAGGTCAAAGGC	1
*egln3*_qP-r	GCAATCCCCCATGTTCCTTGGCA	2-3
*ttll11*_qP-f	TCCCAGCATGAGGATTGAACACG	3
*ttll11*_qP-r	CCGGCATTTGACGTGATGTGTTTC	4-5
*camk2b*_qP-f	AGAGGCCCGGATCTGCCGTT	1
*camk2b*_qP-r	TGCTGGTGGATGTGGCTGACG	4
*btr01*_qP-f	CGGCCATACTGTGACGGAGGC	1
*btr01*_qP-r	CTGAGACCGCAGTTCGGCGG	3
*ncam2*_qP-f	TCGGATTGCTCGTCGGTGGC	1
*ncam2*_qP-r	GCCTGGCACCGGTAGATGCC	3
*crtc3*_qP-f	GCAGCTCATGACAGACCTCACTGT	1
*crtc3*_qP-r	TGGAAATCAGTGCTGGCATTGCCT	2
*irs2*_qP-f	ACACAGCTCTGCCTCCGTAGA	1
*irs2*_qP-r	GGAGTAACCTCTGCTTCCTGCTCA	2
*sept8a*_qP-f	CCTCGGCGGTCATGTTGGCTT	1-2
*sept8a*_qP-r	GTTCTGGGCCGCAGGTAGACG	3
*eef1a1l1*_qP-f	AGAAGGAAGCCGCTGAGATGG	3
*eef1a1l1*_qP-r	TCCGTTCTTGGAGATACCAGCC	4

II. Primers used for amplification and cloning of full-length mRNAs.

**Primer**	**Sequence (5' to 3')**
*irs2*_FL-f: ClaI / **Start**	GGTGGTATCGAT**ATG**GCAAGTCCGCCTCTTA
*irs2*_FL-r: XhoI / **Stop**	GCCACCCTCGAG**TCA**ATCTTGTACAGTGGTTGCAG
*crtc3*_FL-f: Cla / **Start**	GGTGGTATCGAT**ATG**TCTGGATCCCCGGGC
*crtc3*_FL-r: XbaI / **Stop**	GCCACCTCTAGA**CTA**AAGTCTGTCACTGCGAAAGC
*btr01*_FL-f: Cla / **Start**	GGTGGTATCGAT**ATG**TCATTTCCTGGTGAATTCCTGTC
*btr01*_FL-r: XbaI / **Stop**	GCCACCTCTAGA**CTA**TTCTAACCAGCTTGGGTCAGC

[1] Relevant restriction sites are underlined, and start/stop codons are in bold.

III. Primers used for amplification and cloning of in situ hybridization probes. Primers containing restriction sites were directionally cloned into pCS2p+. Primers without restriction sites were TA cloned into vector pCRII and directionality was validated by sequencing.

**Primer**	**Sequence (5' to 3')**
*bsg*_IS-f: BamHI	ATGCGGATCCGGTTTCAAGCCGAAGCTATG
*bsg*_IS-r: XbaI	ATGCTCTAGAGACCCAATAGTGGCCTTTGA
*ythdf2*_IS-f: BamHI	ATGCGGATCCCAAACAAACGCGCAGTCTAA
*ythdf2*_IS-r: XbaI	ATGCTCTAGACAGTGCTCCAGACTGTCCAA
*smarca5*_IS-f: BamHI	ATGCGGATCCGGAGAGCGTTTACGATGAGC
*smarca5*_IS-r: XbaI	ATGCTCTAGACGACAGCACAGCCGTAGTTA
*irs2*_IS-f:	CTTCAGTCAGCCCCACTAAC
*irs2*_IS-r:	CCTGCTTTACAACAACCGCC
*crtc3*_IS-f:	GGACGTTTCCCTCAGGCCTG
*crtc3*_IS-r:	GGTAGTGGGACAGACCCGCG
*btr01*_IS-f:	CGTGCATTTCCTCCTACTGGG
*btr01*_IS-r:	GCAAACATACTGATGGTGCCTG
