# Supplementary material for: Novel Genes Critical for Hypoxic Preconditioning in Zebrafish Are Regulators of Insulin and Glucose Metabolism
Source: G3 (Bethesda). 2015 Apr 3;5(6):1107–16. doi: 10.1534/g3.115.018010 (PMC4478541; doi:10.1534/g3.115.018010)
Supplement: Supporting Information [file supp_g3.115.018010_TableS5.pdf]

Table S5 Full-length cDNA sequence information. Differences between our full-length cDNA sequence and the Ensembl transcript are listed below.

Gene<br>**Reference transcript**	**Location of Base Change**	**Consequence**
irs2<br>ENSDART00000053924	NA	NA
btr01<br>ENSDART00000073689	1129, insertion<br>(45 nucleotides)	retention of 45 nucleotides of predicted intronic sequence at the exon-intron 11 boundary, with a resulting in-frame addition of 15 amino acids:<br>GTTTGCACCCTTCCACAGAA<br>AGATCGCTGGGAGTGTATCA<br>ACAAA
crtc3<br>ENSDART00000073903	435-440, deletion<br>(6 nucleotides)	splicing-mediated exclusion of the first 6 bases of predicted exon 5, with a resulting in-frame deletion of two amino acids:<br>CCACAG
crtc3<br>ENSDART00000073903	G1098A	synonymous variant (proline)
crtc3<br>ENSDART00000073903	1347, insertion<br>(45 nucleotides)	addition of 45 nucleotides from a small exon within intron 4 of the Ensembl gene structure for which there is established EST evidence (AL913705), resulting in the addition of 15 amino acids:<br>GCAGGTGGACAACAACAACA<br>ACAACAGCAGCAACAGCAGC<br>ACCAC
