# Supplementary material for: Novel Genes Critical for Hypoxic Preconditioning in Zebrafish Are Regulators of Insulin and Glucose Metabolism
Source: G3 (Bethesda). 2015 Apr 3;5(6):1107–16. doi: 10.1534/g3.115.018010 (PMC4478541; doi:10.1534/g3.115.018010)
Supplement: Supporting Information [file supp_g3.115.018010_TableS6.pdf]

Table S6 Genes with expression most highly correlated to *egln3* under all conditions, n = 6.

**Transcript**	**EST evidence**	**Gene info**
ENSDART00000028950	XM_684484	*gabrr3*, gamma-aminobutyric acid (GABA) receptor, rho 3
NM_001077375	NM_001077375	*ttll11*, tubulin tyrosine ligase-like family, member 11
OTTDART00000014783	XM_001332019	*pde4c*, phosphodiesterase 4C, cAMP-specific
ENSDART00000073688	XM_686415	*btr01*, bloodthirsty-related gene family, member 1
OTTDART00000008953	OTTDART00000008953	*msrb3*, methionine sulfoxide reductase B3
ENSDART00000064860	XM_682340	*rbms2*, RNA binding motif, single stranded interacting protein
